# Habitat and climate shape growth patterns in a mountain ungulate

**DOI:** 10.1002/ece3.8650

**Published:** 2022-03-07

**Authors:** Rudolf Reiner, Andreas Zedrosser, Hubert Zeiler, Klaus Hackländer, Luca Corlatti

**Affiliations:** ^1^ 27270 Berchtesgaden National Park Berchtesgaden Germany; ^2^ Institute of Wildlife Biology and Game Management University of Natural Resources and Life Sciences, Vienna Vienna Austria; ^3^ Department of Natural Sciences and Environmental Health University of South‐Eastern Norway Telemark Norway; ^4^ Deutsche Wildtier Stiftung (German Wildlife Foundation) Hamburg Germany; ^5^ 9174 Wildlife Ecology and Management University of Freiburg Freiburg Germany; ^6^ 9174 Stelvio National Park Bormio Italy

**Keywords:** body mass, chamois, climate change, growth pattern, *Rupicapra rupicapra*, ungulate

## Abstract

Uptake and use of energy are of key importance for animals living in temperate environments that undergo strong seasonal changes in forage quality and quantity. In ungulates, energy intake strongly affects body mass gain, an important component of individual fitness. Energy allocation among life‐history traits can be affected by internal and external factors. Here, we investigate large‐scale variation in body growth patterns of Alpine chamois *Rupicapra rupicapra rupicapra*, in relation to sex, age, temperature, and habitat variations across 31 (sub)populations in the Central European Alps. Taking advantage of an exceptionally large dataset (*n* = 178,175) of chamois hunted over 27 consecutive years between 1993 and 2019 in mountain ranges with different proportions of forest cover, we found that (i) patterns of body mass growth differ between mountain ranges, with lower body mass but faster mass growth with increasing proportion of forest cover and that (ii) the effect of spring and summer temperatures on changes in body growth patterns are larger in mountain ranges with lower forest cover compared to mountain ranges with higher forest cover. Our results show that patterns of body mass growth within a species are more plastic than expected and depend on environmental and climatic conditions. The recent decline in body mass observed in Alpine chamois populations may have greater impacts on populations living above the treeline than in forests, which may buffer against the effects of increasing temperatures on life‐history traits.

## Graphical abstract

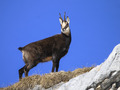

## 1 INTRODUCTION

Body mass reflects the overall energy stored in an individual and is a good proxy for individual fitness (Douhard et al., 2017; Plard et al., 2015; Wilder et al., 2016). For animals living in temperate environments that undergo strong seasonal changes in forage quality and quantity, the cyclic uptake and use of energy profoundly affects key life‐history traits (Mautz, 1978; Sparling et al., 2006). Energy allocation of acquired resources face life‐history trade‐offs, as different fractions of the overall energy‐budget are invested into survival and reproduction (Stearns, 1989, 1992), as observed in many taxa, including mammals (Bergqvist et al., 2018; Hamel, Garel et al., 2009; Ozgul et al., 2010), birds (Chastel et al., 1995), and insects (Akman & Whitman, 2008; Sturm, 2016). In ungulates, energy intake strongly affects body mass gain (Parker et al., 2009), which is an important component of individual fitness (Gaillard et al., 2000). In turn, body mass is strongly affected by internal factors such as sex and age, as well as by environmental conditions, thus it is expected that trade‐offs among life‐history traits are also affected by sex, age, and variations in habitat types and climatic conditions (Blanckenhorn, 2000).

In large herbivores, sex‐specific differences in energy allocation to body mass primarily result from sexual selection (Loison, Gaillard et al., 1999). With respect to age variation, the energy‐allocation trade‐off appears to be more complex in adult individuals than in subadults, as the former not only have to allocate the available resources into several somatic traits such as growth and survival but also into reproduction (Gibbens & Arnould, 2009; Toïgo et al., 2007). Numerous studies suggest that energy allocation for reproduction peaks at prime‐age stage when body growth is largely completed (Mainguy & Côté, 2008; Yoccoz et al., 2002). In old individuals, when resources are increasingly invested in survival because of physiological senescence, energy allocation into reproduction typically decreases for most species (Festa‐Bianchet et al., 1998; Mason et al., 2011; Morin et al., 2016). Nonetheless, owing to greater experience and larger body mass, the decreasing energy allocation of old females into reproduction does not necessarily translate into lower reproductive success (Beauplet et al., 2006; Green, 1990; Hamel et al., 2010), and in some species best reproducers tend to live longer (*cf*. Toïgo et al., 2013).

Furthermore, environmental features such as habitat and climatic conditions may strongly affect body growth patterns via different mechanisms (Forchhammer et al., 2001; Pelletier et al., 2007). For example, individuals living in predictable and/or favorable environmental conditions, e.g., in habitats that provide sufficient food and are protected against extreme climatic variation, commonly show lower mortality rates and invest earlier into reproduction than into body growth (Bleu et al., 2015; Clutton‐Brock et al., 1996). In comparison, individuals living in harsher and/or unpredictable environmental conditions commonly allocate fewer resources into early reproduction but more into somatic growth and ultimately survival (Bårdsen et al., 2011; Herfindal et al., 2006). Individuals living in harsh environmental conditions therefore need to survive to an older age to reach the same lifetime reproductive success, compared with individuals living in more favorable conditions (Hamel, Gaillard et al., 2009; Krüger, 2002; McLoughlin et al., 2007). In addition, differences in forage conditions, both in terms of quality and quantity, may result in body mass differences across altitudinal and latitudinal gradients (Mysterud et al., 2001; Ronget et al., 2018). Short summers, typical of high altitudes and latitudes, are assumed to favor fast‐growing plants, which in turn are associated with high forage quality (Bliss, 1962; Mysterud et al., 2001; Van Soest, 1994) and result in greater body mass of herbivores (Geist, 1987; Herfindal et al., 2006; Sand et al., 1995). The ability to migrate and follow the “green wave” of new growth along an altitudinal gradient may lead to heavier body masses in regions with low forest cover which are also typically associated with greater altitudinal range (Bischof et al., 2012; Merkle et al., 2016; Middleton et al., 2018). Besides spatial variation in the context of habitat characteristics, also population density – due to increased competition for food resources (Mysterud et al., 2002; Pettorelli et al., 2001) – and changing climatic conditions are known to affect body growth patterns of numerous species (LeBlanc et al., 2001; Ozgul et al., 2010; Rode et al., 2010; Yom‐Tov, 2001). Internal and external factors may also interact to shape variations in body mass patterns.

**FIGURE 1:**
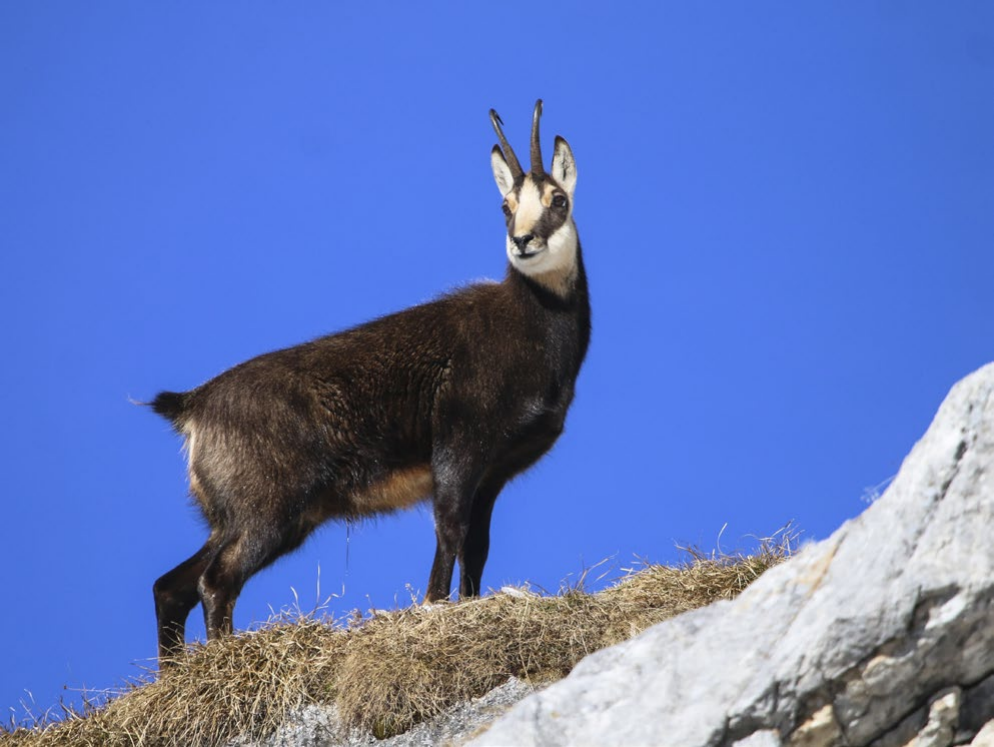
The Alpine chamois *Rupicapra rupicapra* is one of the most iconic mammals of mountainous regions of Europe and is inhabiting alpine prairies as well as montane and subalpine forests throughout the Alps

In polygynous and sexually dimorphic species, temperature‐induced variation in body mass typically is more pronounced in males than in females, especially in younger age classes (Pérez‐Barbería et al., 2020; Rughetti & Festa‐Bianchet, 2012). Males in such species may therefore be more vulnerable to variations in climatic conditions compared to females, as their mass growth requires more energetic resources and lasts longer until fully grown (e.g., in red deer *Cervus elaphus*: Clutton‐Brock et al., 1982). In adults of such species, females’ body mass is mostly limited by forage resources and affected by pregnancy, parturition, and lactation, while male body mass is affected by forage resources as well as male–male competition for reproduction (Clutton‐Brock, 1988; Ritchot et al., 2021). To date, very few studies have evaluated the spatial variation in the context of environmental conditions in the differences of body growth patterns between sexes (McLellan, 2011; Swenson et al., 2007), although there is some evidence that spatial variation may be more pronounced in males than in females, for example, in Alpine chamois *Rupicapra rupicapra rupicapra* (Mason et al., 2014) (Figure 1).

The Alpine chamois is the most abundant mountain ungulate of the European Alps (Corlatti et al., 2011) and inhabits alpine areas above the tree line as well as subalpine and montane forests (*cf*. Reiner et al., 2021). Male chamois have a longer period of body growth (Garel et al., 2009) and show greater intra‐annual variations in mass than females due to higher mass loss during the rut (Mason et al., 2011). Compared to other mountain ungulates (e.g., Alpine ibex *Capra ibex*: Polák & Frynta, 2009; bighorn sheep *Ovis canadensis*: LeBlanc et al., 2001), sexual size dimorphism in chamois is weak (Rughetti & Festa‐Bianchet, 2011). Recent studies suggest that key life‐history traits, such as survival and reproductive effort, vary substantially between chamois populations (Bleu et al., 2015; Mason et al., 2011). Chamois are morphologically and physiologically adapted to environmental conditions at high altitudes, i.e., cold winters with high precipitation in the form of snow (Ascenzi et al., 1993); therefore, spatial and temporal variations in body mass in context with increasing temperatures may occur (Ciach & Pęksa, 2018; Reiner et al., 2021; Rughetti & Festa‐Bianchet, 2012; Willisch et al., 2013). Changes in environmental conditions may affect patterns of mass and mass growth differently in habitats with different characteristics. For example, Reiner et al. (2021) showed that, in chamois, the effects of increasing spring and summer temperatures on yearling body mass are mitigated in forested areas, which suggests a temperature‐buffering effect of forests. These habitat‐specific differences in body mass are likely related to differences in foraging opportunities and thermoregulatory costs (Bubenik, 1984).

Here we investigate large‐scale variations in the patterns of body mass and growth of Alpine chamois in relation to increasing spring and summer temperatures and habitat variations (i.e., proportion of forest cover), while controlling for the effects of sex, age, and population density across 31 (sub)populations in the Central European Alps. We take advantage of an exceptionally large dataset (*n* = 178,175) of chamois harvested over 27 consecutive years between 1993 and 2019 in mountain ranges with different proportions of forest cover (Reiner et al., 2021). Specifically, we hypothesize (i) a slower growth in mass and higher adult mass in mountain ranges with less predictable environmental conditions (i.e., alpine areas with lower forest cover) and (ii) larger differences in body mass patterns in relation to variations in spring and summer temperatures in mountain ranges with lower forest cover compared to mountain ranges with higher forest cover (Cook et al., 1998; Reiner et al., 2021).

## 2 MATERIALS AND METHODS

### 2.1 Study area and populations

**FIGURE 2:**
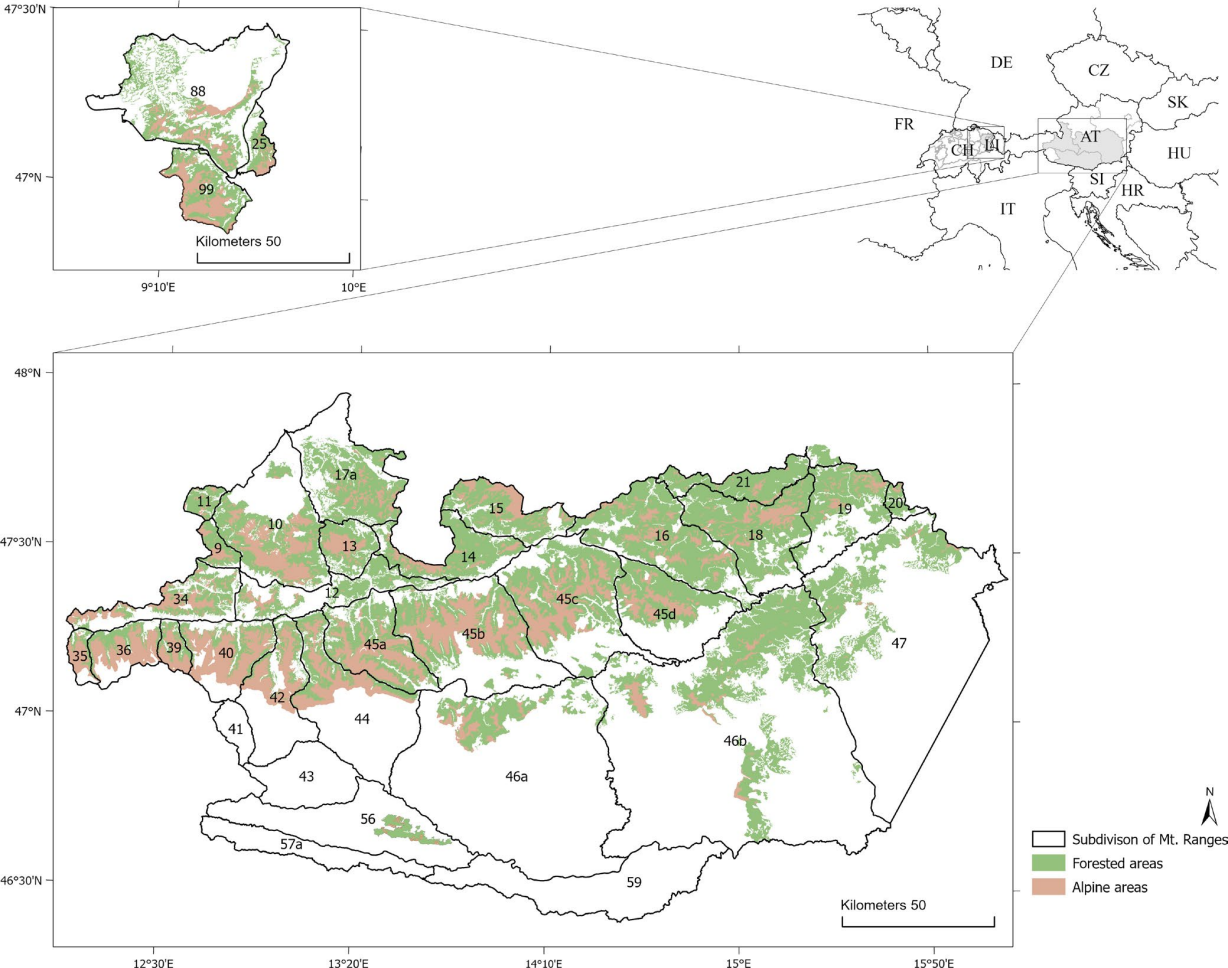
Location of the study area in Austria (in the provinces of Carinthia, Salzburg, and Styria), in Switzerland (in St. Gall Canton), in Liechtenstein, and in Germany (in the State of Bavaria) to evaluate the effects of habitat and climate on body mass in Alpine chamois, 1993–2019. Numbers and black lines correspond to the geographical subdivision of the Eastern Alps. Mountain range IDs between 9–25 belong to the Northern Limestone Zone, 35–47 to the Central Alps, 56–59 to the Southern Limestone Zone, and 88–99 to the Western Alps. Colored areas show suitable habitat for chamois within hunting management units, i.e., the sum of all open Alpine areas (i.e., Alpine meadows, sparsely vegetated areas, and bare rocks) and all forested areas (i.e., broadleaf, coniferous, and mixed forests) in all hunting management units with chamois harvest during the study period

We studied 31 Alpine chamois (sub)populations, of which were 28 in Austria (in the provinces of Carinthia, Salzburg, and Styria) (*cf*. Reiner et al., 2021), two in Switzerland (in Canton of St. Gall), one in Liechtenstein, and one in Germany (in the State of Bavaria) (Figure 2). The overall study area covers ~13,350 km² and includes all hunting areas (i.e., game management units of at least 1.15 km²) where chamois have been harvested in the provinces of Salzburg and Styria, in Canton St. Gall, and in Liechtenstein, as well as some hunting areas in Carinthia and in Bavaria. Hunting areas were grouped into populations based on the mountain range they were located in, following the geographical subdivision of the Alps (Grassler, 1984) (Appendix S1: Table S1). The grouping according to mountain ranges coincides with chamois (sub)populations (*n* = 31) with only limited dispersal among (sub)populations (*cf*. Mason et al., 2011; Reiner, 2015; Reiner et al., 2020; see Reiner et al., 2021 for a detailed description of the study area and the geographical subdivision).

### 2.2 Chamois data

Regional and national hunting organizations and game management authorities collected data (for further details see Reiner et al., 2020 or Reiner et al., 2021) on body mass (eviscerated, without head but with skin, at a precision of 0.5 kg), sex, age (in years), hunting area, and date shot of 178,175 chamois (87,825 females, 90,350 males) harvested between 1993 and 2019 (1993–2019 in Styria, Carinthia, Liechtenstein, and Bavaria; 1996–2019 in St. Gall; 1998–2019 in Salzburg; Appendix S1: Table S2). Age was determined by counting horn growth annuli (Corlatti, Gugiatti et al., 2015; Schröder & von Elsner‐Schack, 1985). Female ages ranged between 1–24 years and male ages between 1–23 years. Date shot was converted to Julian day and ranged between day 197 (16th July) and 365 (31st December) (days 198–366 for leap years). Body mass was adjusted for variations in shooting date to day 301 (28 October or 27 October in leap years; median date shot and approximated start of rut) by fitting quadratic linear regression models between body mass and Julian date for each sex and age. Since most chamois are born in late May and early June (Kourkgy et al., 2016; Perez‐Barberia et al., 1996), we used the age of individuals as 1.5, 2.5, 3.5 years, etc., to model growth and throughout the text. To account for density‐dependent variation in body mass, we used the annual number of harvested chamois in relation to suitable habitat within each mountain range (i.e., forests and open alpine areas in hunting areas with chamois harvest) as a density index (Reiner et al., 2021).

### 2.3 Forest cover data

To investigate differences in growth patterns among habitats, we calculated the area of different habitat types in each hunting area (see Reiner et al., 2021) based on Corine Landcover data (Copernicus Land Monitoring Service, 2018) in ArcGIS Pro 2.8 (ESRI Inc., 2021). Therefore, the proportion of area covered by forests (broad‐leaved, coniferous, mixed forests) in relation to open alpine areas (alpine meadows, sparsely vegetated areas, bare rocks) was calculated for every hunting area with chamois harvest for each mountain range during the study period (Reiner et al., 2021). We used the relative area covered by forest within each mountain range (hereafter “forest cover”) as predictor variable in further analyses (Reiner et al., 2021). Mean forest cover of mountain ranges was 70.2%, ranging from 24.1% to 99.0% (Appendix S1: Table S1).

### 2.4 Climatic data

We obtained climatic data to investigate if body growth patterns vary between climatic subperiods of different spring and summer temperatures (“cold” vs. “warm” subperiod) from the Central Institution for Meteorology and Geodynamics (Austria), Federal Office of Meteorology and Climatology (Switzerland), Office for Environment (Liechtenstein), and the German Meteorological Service (Germany). We calculated the mean daily maximum spring (April–May) and summer (June–August) temperatures (in °C) for each year during the study period. We then tested for periods with statistically different spring and summer temperatures by dividing the overall study period into 2–5 climatic subperiods and calculating the Calinski‐Harabasz (CH) index (Caliński & Harabasz, 1974) for all possible chronological combinations of climatic subperiods (Gosselin et al., 2015). The CH index is calculated as [trace *B*/(*k*−1)]/[trace *W*/(*n*−*k*)], where *n* is the total number of elements and *k* is the number of clusters in the solution. The terms *B* and *W* are the sum of the squares between and within clusters and the cross product matrices, and the trace is the sum of the main diagonal of the matrices (Caliński & Harabasz, 1974; Milligan & Cooper, 1985). Higher values of the CH index mean a higher between‐cluster variance relative to within‐cluster variance. We compared the CH index for the most likely chronological groups and gave the most probable number of sub‐periods. The maximum level of hierarchy was used to determine the correct number of partitions in the data that would maximize the inter‐cluster variance and minimize the intra‐cluster variance, i.e., return periods with different spring and summer temperatures (Gosselin et al., 2015).

### 2.5 Statistical analysis

To explore the variation in mass growth patterns in relation to different proportions of forest cover, we fitted a “global” Gaussian linear mixed models (LMM) for each sex, with body mass as the response variable. Population density (as covariate) and the interaction between age^2^ and forest cover were included as explanatory variables. Mountain range (*n* = 31) and year (*n* = 27) were fitted as random intercepts. This random structure allowed us to consider regional differences as well as to account for correlation among data within the same mountain range (Davies et al., 2006; Dormann et al., 2007). To compare the length of the period of mass growth, we used the age when 99% of maximum adult body mass was achieved (age_99%_) from fitted models for each sex (Garel et al., 2006, 2009; Sand et al., 1995).

To address the second hypothesis, i.e., differences in mass growth patterns between climatic subperiods (“cold” *vs*. “warm” subperiod) are larger with decreasing forest cover, we compared mass growth rates in different climatic subperiods. We therefore fitted Gaussian linear mixed models (LMM), with body mass as the response variable and added an interaction with climatic subperiod to the models, based on the CH index, resulting in the 3‐way interaction: age^2^ × forest cover × climatic subperiod. In chamois, males and females have different patterns of body mass growth across age (Garel et al., 2009). To account for this sex‐specific patterns while avoiding the complexity of a 4‐way interaction, sexes were modeled separately. Population density was fitted as a covariate. For all models, we checked for normality and homogeneity of the conditional distribution by inspecting standardized residuals against fitted values (Zuur et al., 2009). To illustrate age and habitat‐specific change in mass between the climatic subperiods, we calculated the difference in body mass between the climatic subperiods for different proportions of forest cover. Confidence intervals (CI; 2.5% and 97.5% quantiles) were calculated using 1000 bootstrap replicates from the fitted models for each sex. All statistical analyses were performed in R 3.6.1 (R Core Team, 2020) via R Studio 1.2.5001 (RStudio Team, 2019). The packages “*splines*” (R Core Team, 2020) and “*lme4”* (Bates et al., 2015) were used for fitting LMMs with non‐linear effects. Marginal effects were visualized using the package “*ggplot2*” (Wickham, 2016). To investigate whether results are related to different distributions of data over time, we checked if results changed when just the period from 1998 to 2019 was considered in the analyses (i.e., years where data for all mountain ranges were available).

## 3 RESULTS

### 3.1 Climatic subperiods of consistent spring and summer temperatures

**FIGURE 3:**
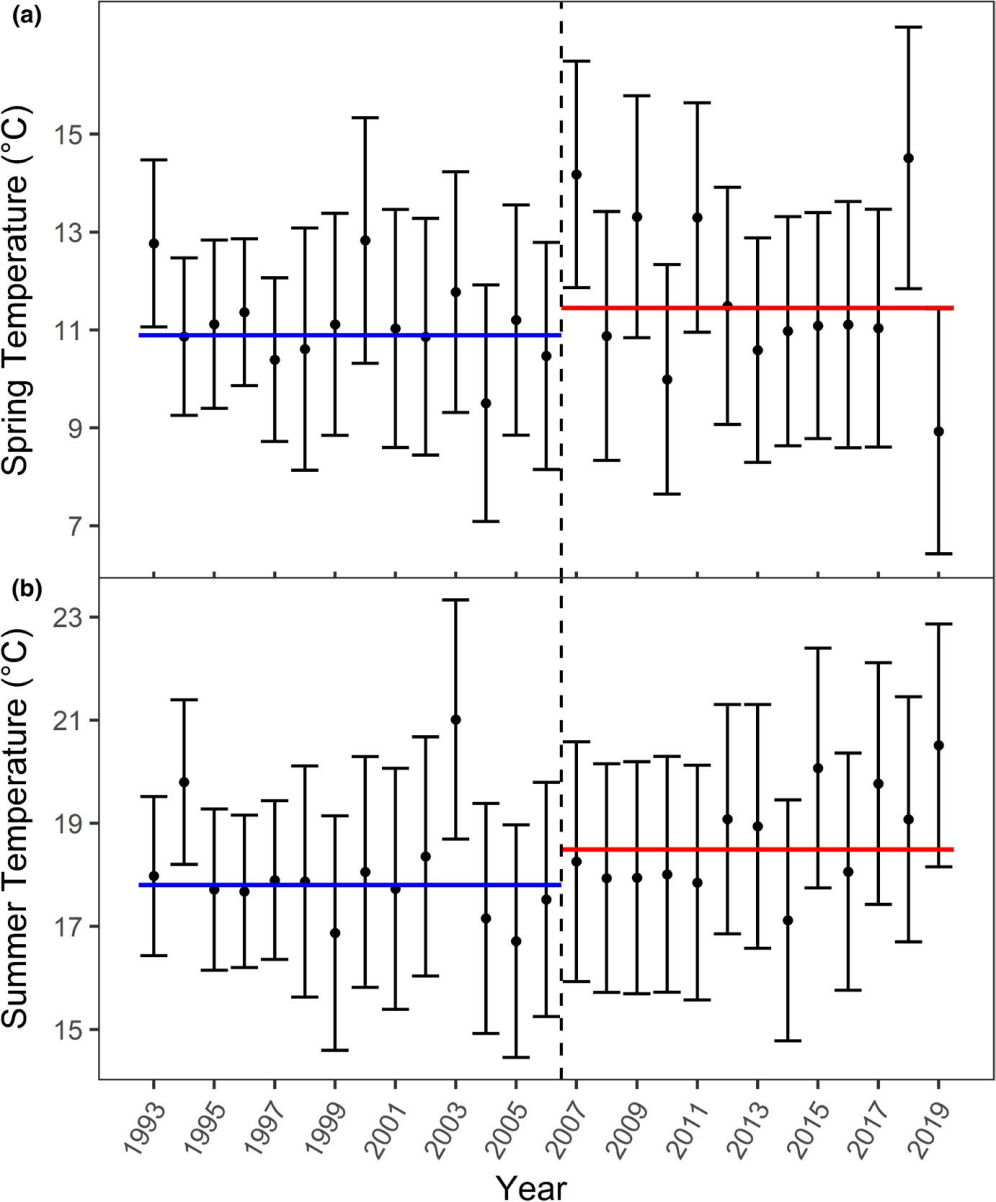
Mean spring (April–May) temperatures (a), and mean summer (June–August) temperatures (b) in 31 mountain ranges in the study area to evaluate the effects of habitat and climate on body mass in Alpine chamois in Austria, Germany, Switzerland, and Liechtenstein, 1993–2019. The dashed line separates the study period into a cold (1993–2006) and a warm (2007–2019) subperiod. The horizontal lines indicate the average temperature in the cold subperiod 1993–2006 (blue) and the warm subperiod 2007–2019 (red). Bars indicate standard deviation

Based on the CH index, the most likely number of climatic subperiods was two (Appendix S1: Table S4). Intra‐group variation was minimized, and inter‐group variation maximized with the two climatic subperiods 1993–2006 (*n* = 14 years), with lower spring (mean ± SE: 10.89°C ± 0.23), and summer temperatures (17.80°C ± 0.28), and 2007–2019 (*n* = 13 years), with higher spring (11.45°C ± 0.44) and summer temperatures (18.49°C ± 0.29) (Figure 3). We therefore retained two climatic subperiods (hereafter: “cold” vs. “warm”) for further analyses. Some of the chamois harvested in the warm subperiod were born in the cold period (before 2007), thereby experiencing cold spring and summer temperatures in early life. Because in chamois at least 85% of the adult body mass is attained by 4.5 years of age (Bassano et al., 2003; Garel et al., 2009), we used 3.5 years as the upper threshold for the consequential long‐term influence of temperature on body mass. We thus excluded all individuals born prior to 2004 from the subperiod 2007 to 2019. Individuals harvested in the subperiod 2007–2019 therefore had a maximum age of 15.5 years. To be consistent we also excluded all animals >15.5 years (*n* = 809) from subperiod 1993 to 2006. Given these criteria, our final sample size was 72,558 females (48,026 in subperiod 1993–2006 and 24,532 in 2007–2019) and 80,659 males (49,667 in subperiod 1993–2006 and 30,992 in 2007–2019).

### 3.2 Patterns of mass growth

**FIGURE 4:**
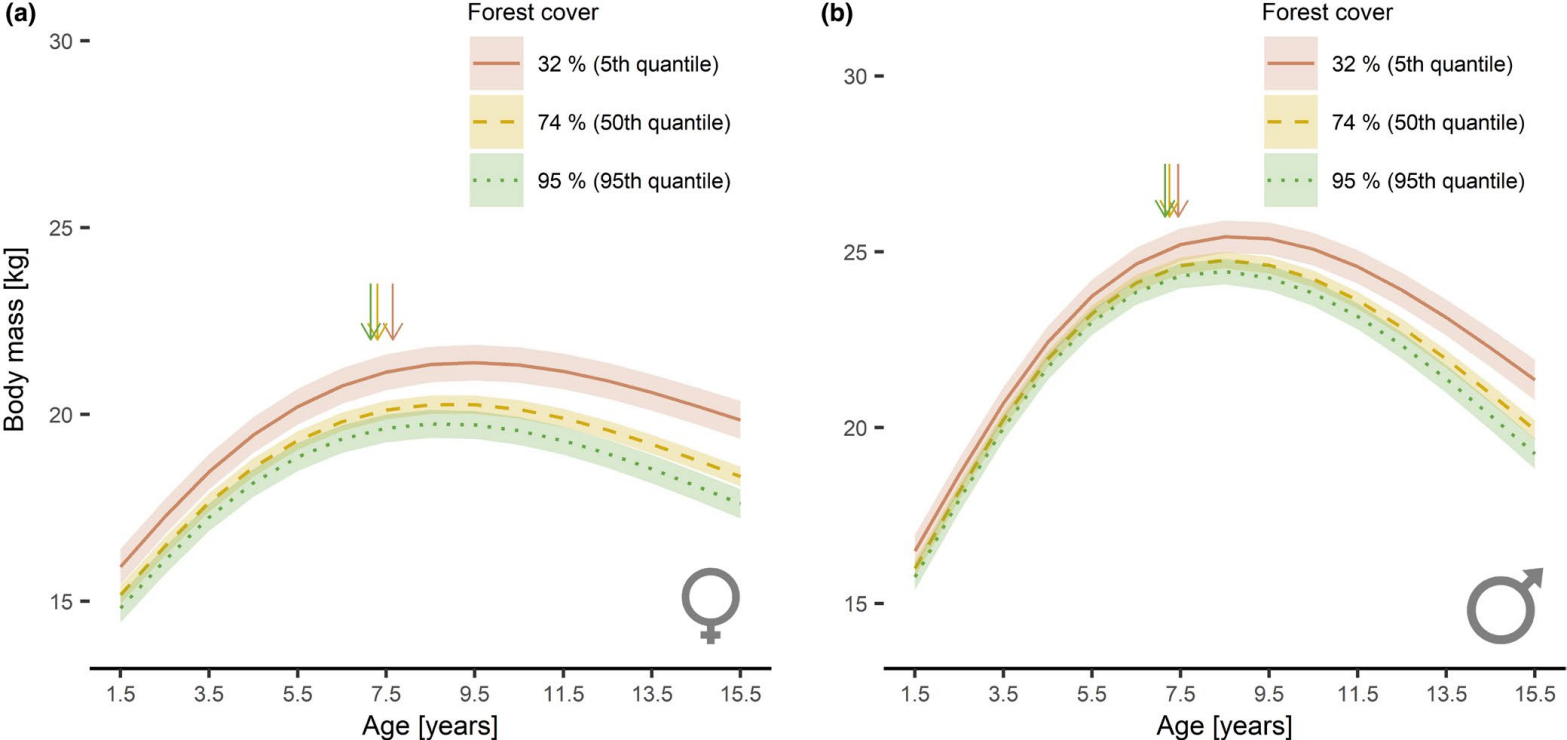
Estimated growth patterns of Alpine chamois females (a) and males (b) harvested in Austria, Germany, Switzerland, and Liechtenstein from 1993 to 2019, as a function of the interaction between age and forest cover. The levels of forest cover correspond to the 5th (32% forest cover; solid red line), 50th (74% forest cover; dashed yellow line), and 95th (95% forest cover; dotted green line) quantiles. Shaded areas indicate the 95% confidence intervals. Arrows indicate estimated age at which animals reach 99% of the estimated peak body mass (i.e., end of the period of active body growth) for the respective proportional forest cover (at 32% forest cover – red arrow; at 74% forest cover – yellow arrow; at 95% forest cover – green arrow)

The model fitted to investigate mass growth patterns of females in relation to different proportions of forest cover showed a significant negative effect of density (*β* = −0.03, 95% CI: −0.05 to 0.00, *p* = .024) and a significant negative interaction age^2^ × forest cover (*β* = −1.64, 95% CI: −2.16 to −1.13, *p* < .001). Similarly, for males the model returned a significant negative effect of density (*β* = −0.06, 95% CI: −0.08 to −0.03, *p* < .001) and a significant negative interaction age^2^ × forest cover (*β* = −2.22, 95% CI: −3.12 to −1.32, *p* < .001). The estimates of the interactive effects in both sexes suggest differences in mass growth patterns in relation to forest cover, i.e., faster growth of mass but lower overall mass with increasing forest cover for both sexes (Figure 4). The estimated age_99%_ decreased with increasing forest cover and ranged from 7.15 years in mountain ranges with 95% forest cover (95th quantile) to 7.65 years in mountain ranges with 32% forest cover (5th quantile) in females and from 7.15 to 7.45 years in males, thereby suggesting that 99% body mass is attained earlier in forested areas than in alpine areas (Figure 4).

**FIGURE 5:**
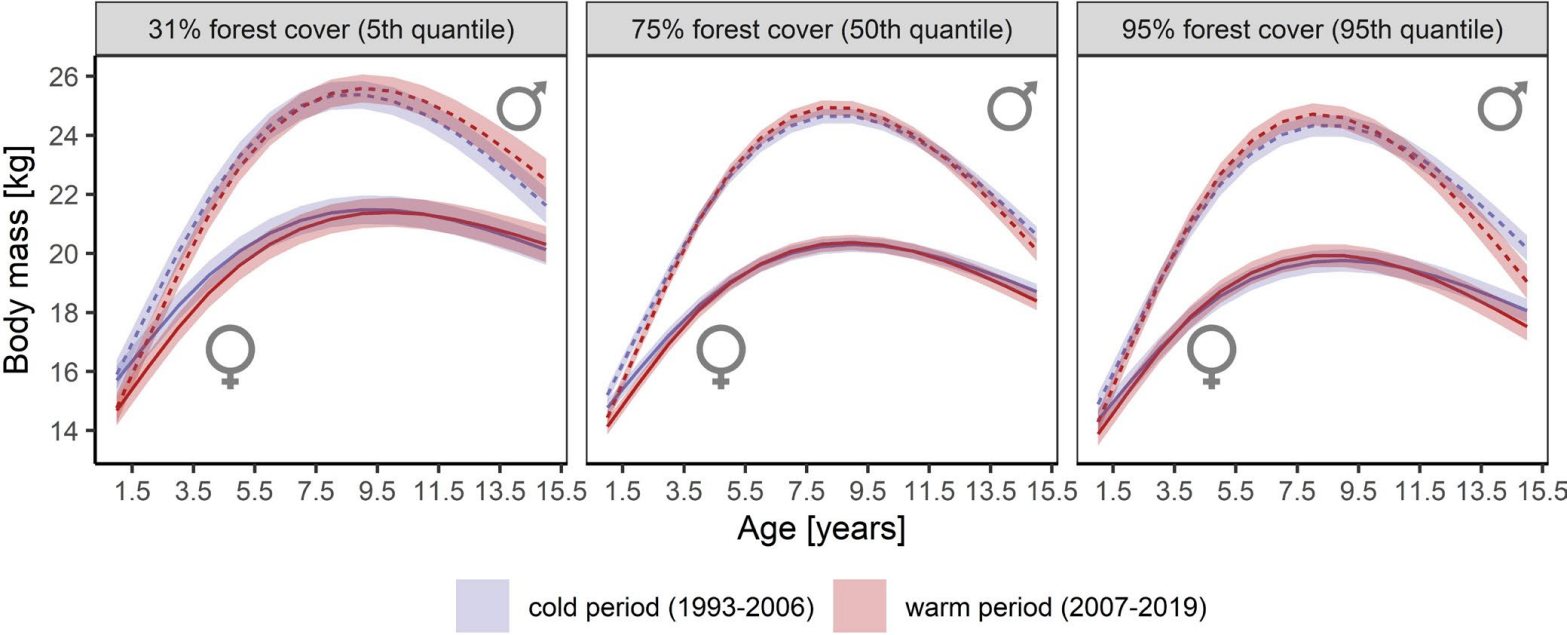
Estimated growth patterns of female (solid lines) and male (dashed lines) Alpine chamois harvested in Austria, Germany, Switzerland, and Liechtenstein, for different levels of proportional forest cover, as a function of the interaction between age and two climatic subperiods: 1993–2006 with lower spring and summer temperatures (blue), and 2007–2019 with higher spring and summer temperatures (red). Shaded areas indicate 95% confidence intervals

**FIGURE 6:**
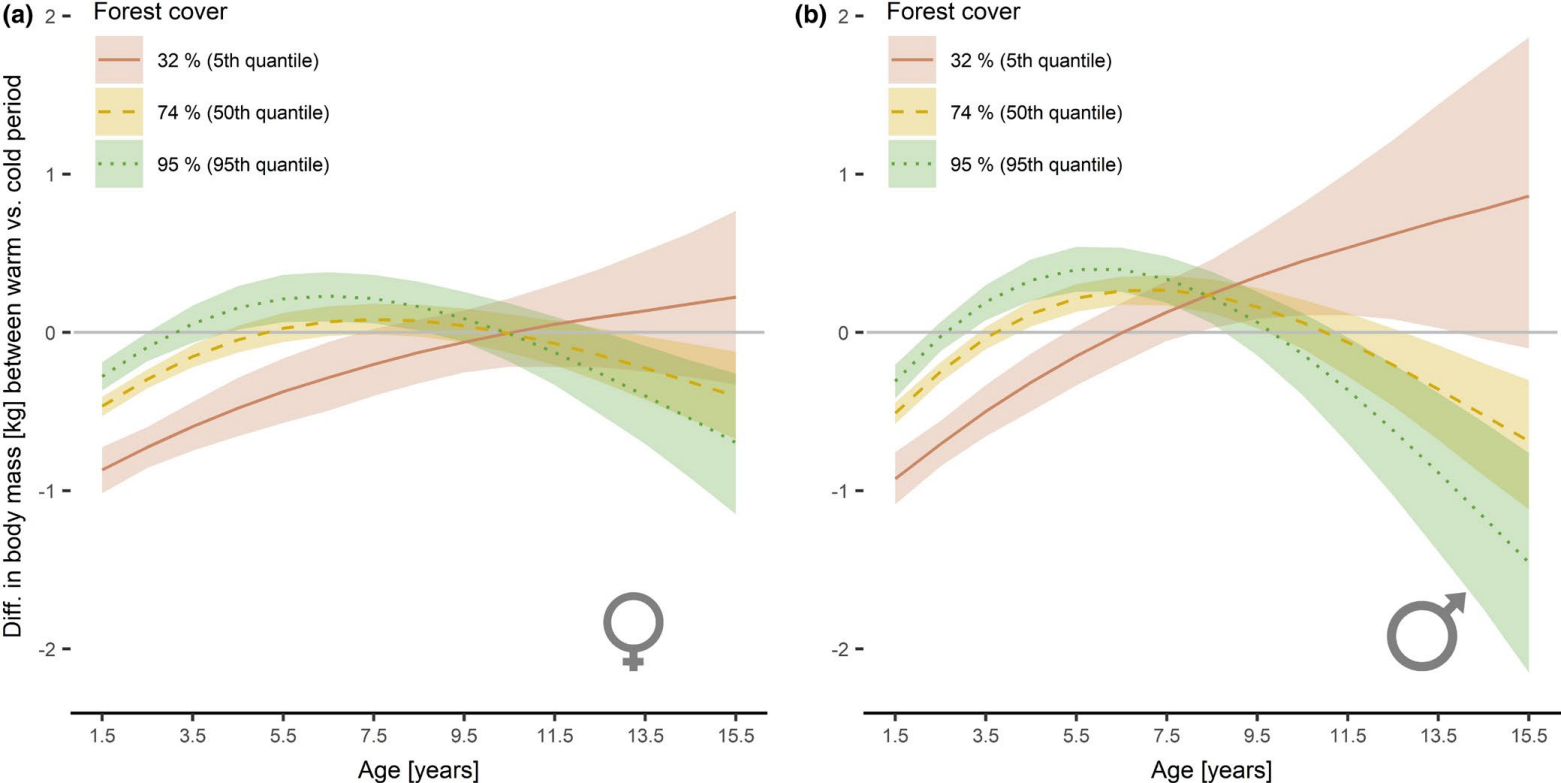
Estimated differences in growth curves between the two climatic subperiods 1993–2006 with lower spring and summer temperatures and 2007–2019 with higher spring and summer temperatures for female (a) and male chamois (b) harvested in Austria, Germany, Switzerland, and Liechtenstein, 1993–2019. The levels of forest cover correspond to the 5th (32% forest cover; solid red line), 50th (74% forest cover; dashed yellow line), and 95th (95% forest cover; dotted green line) quantiles. The grey horizontal line indicates zero difference in body mass between the periods. Values below zero indicate lower body mass in the “warm” period 2007–2019 compared to the “cold” period 1993–2006 for the corresponding age. Shaded areas indicate 95% confidence intervals

Overall mean body mass across all ages in the cold subperiod was 18.3 ± 3.5 kg in females and 21.1 ± 4.6 kg in males but declined during the warm subperiod by 1.0 kg (5.5%) to 17.3 ± 3.6 kg for females and by 0.8 kg (3.8%) to 20.3 ± 3.6 kg for males (Appendix S1: Table S3). When adding the 3‐way interaction “age^2^ × forest cover × climatic subperiod” to the model, the estimates for the interactive effect for females (*β* = −2.32, 95% CI: −3.58 to −1.05, *p* < .001) and males (*β* = −4.59, 95% CI: −6.55 to −2.63, *p* < .001) suggest a difference in the mass growth patterns between climatic subperiods as a function of forest cover, with increasing differences between the climatic subperiods with decreasing forest cover, i.e., in areas with a lower proportion of forest cover, the mass of both sexes was greater during the first years of life in the cold subperiod compared to the warm subperiod (Figure 5). This pattern disappears in mountain ranges with higher proportional forest cover; however, the average body mass in these areas is generally lower than compared to mountain ranges with lower forest cover (Figures 5 and 6).

**FIGURE 7:**
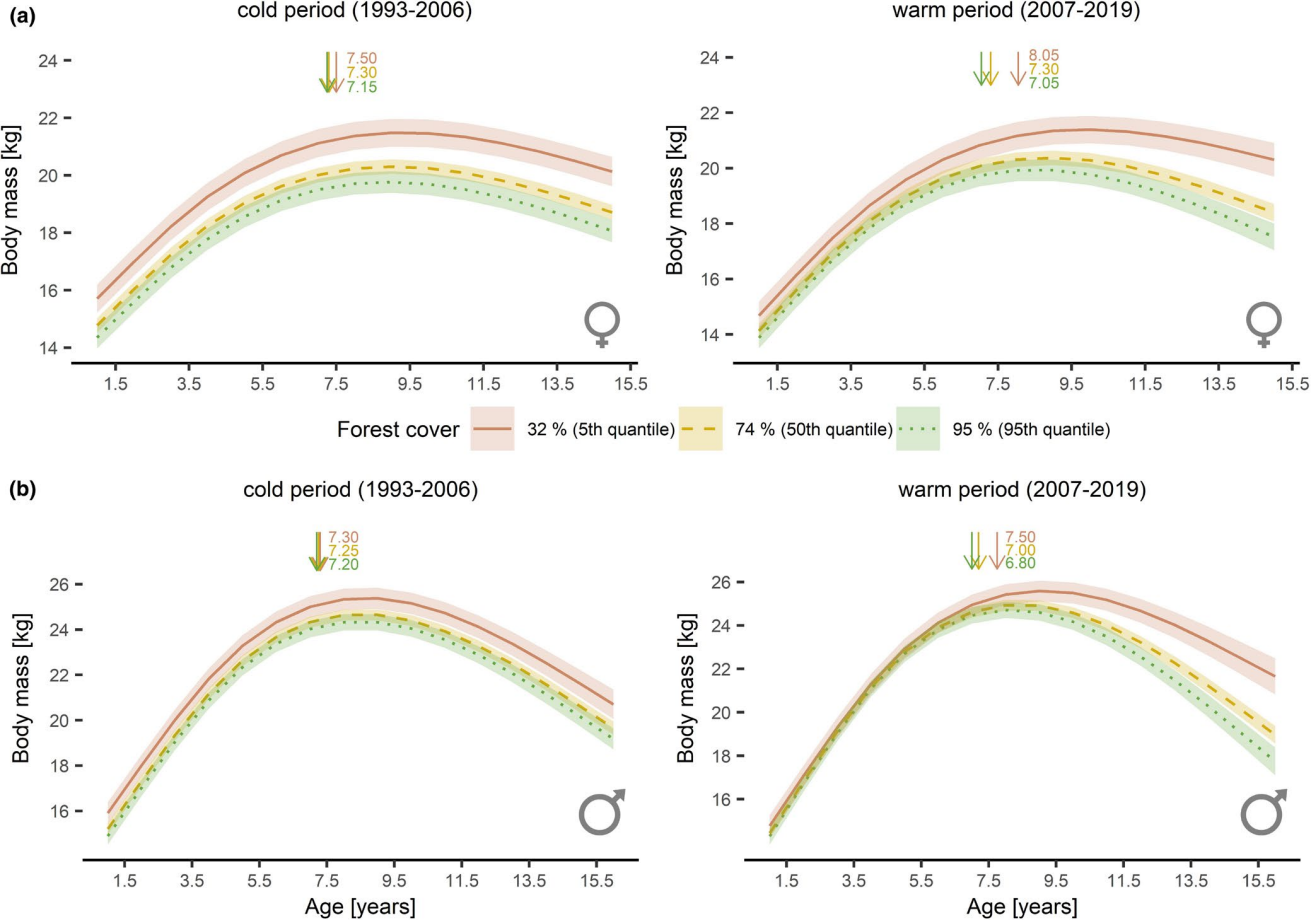
Estimated growth patterns of Alpine chamois females (a) and males (b) harvested in Austria, Germany, Switzerland, and Liechtenstein between 1993–2006 (left) and 2007–2019 (right) as a function of the interaction between age and proportional forest cover. The levels of forest cover correspond to the 5th (32% forest cover; solid red line), 50th (75% forest cover; dashed yellow line), and 95th (95% forest cover; dotted green line) quantiles. Shaded areas indicate the 95% confidence intervals. Arrows and numbers indicate the estimated age at which chamois reach 99% of the estimated peak body mass (i.e., end of period of active body growth) in relation to the respective proportional forest cover

For females, age_99%_ in mountain ranges with 95% forest cover was at 7.25 years in the cold subperiod and 7.05 years in the warm subperiod while in mountain ranges with 32% forest cover it was 7.50 years in the cold subperiod and 8.05 years in the warm subperiod. For males, age_99%_ was 7.20 years in the cold subperiod and 7.00 years during the warm subperiod in mountain ranges with 95% forest cover. In mountain ranges with 32% forest cover, it was 7.30 years in the cold subperiod and 7.75 years in the warm subperiod (Figure 7).

Mean estimated body mass across all ages decreased with increasing forest cover. In forested mountain ranges (95% forest cover), it was 18.3 ± 1.6 kg for females and 21.8 ± 2.5 kg for males in the cold subperiod. For females, it remained stable in the warm subperiod at 18.3 ± 1.8 kg, but mass decreased in males by 0.2 kg (0.9%) to 21.6 ± 2.8 kg. In mountain ranges with low proportional forest cover (32%), it was 20.0 ± 1.8 kg for females and 22.9 ± 2.5 kg for males in the cold subperiod. Mass decreased by 0.3 kg (1.5%) to 19.7 ± 2.1 kg for females and remained stable at 22.9 ± 2.9 kg for males in the warm subperiod (Figure 5). These results (both in terms of subdivision into climatic subperiods and estimated significances) did not change when carrying out the same analysis for the period 1998–2019 (Appendix S1: Table S4 & Appendix S1: Table S5).

## 4 DISCUSSION

The results show that the patterns of body mass of female and male Alpine chamois are affected by variations in the habitat, i.e., the proportion of forest cover within a mountain range: chamois are generally heavier in mountain ranges with lower forest cover and reach peak body mass later compared to mountain ranges with higher forest cover. These findings confirm hypothesis (i), i.e., slower mass growth and higher adult body mass in both sexes in areas with lower forest cover. Furthermore, the significant interaction between age, forest cover, and the climatic subperiods supports hypothesis (ii) for both sexes, i.e., larger differences in body mass in relation to variations in spring and summer temperatures in mountain ranges with lower forest cover compared to mountain ranges with higher forest cover. For females ≤10.5 years and males ≤7.5 years, the decline in body mass during the warm period was larger in mountain ranges with lower forest cover compared to mountain ranges with higher forest cover (Figure 6). A contrasting pattern was observed for old individuals: females >10.5 years and males >7.5 years showed a greater decline in body mass with increasing forest cover during the warm period, while our results suggest no decline in the body mass of old individuals in open alpine areas (Figure 6).

Body mass varies in relation to environmental conditions in ungulates, for example, in moose *Alces alces* (Herfindal et al., 2006, 2020), roe deer *Capreolus capreolus* (Pettorelli et al., 2003; Toïgo et al., 2006), and reindeer *Rangifer tarandus* (Tveraa et al., 2013). Most studies suggest heavier individuals with increasing altitude and latitude (Ericsson et al., 2002; Herfindal et al., 2014), where typically short but intense summers are followed by long and harsh winters (Herfindal et al., 2006; *cf*. Morin et al., 2016). The negative relationship between body mass and the proportion of forest cover we found in chamois may be explained by better vegetation quality and environmental phenology in open alpine areas compared to more forested areas (Albon & Langvatn, 1992; Post & Stenseth, 1999; Van Soest, 1994). Large differences in elevation, typically associated with alpine regions, allow herbivores to migrate and take advantage of the "green wave" of growth over a longer period (Bischof et al., 2012; Merkle et al., 2016; Middleton et al., 2018). Winter severity, which is typically more pronounced at high altitudes with low forest cover, is also assumed to favor large individuals (Loison, Langvatn et al., 1999; Sæther et al., 1996). A possible explanation for this may be found in the lower natural mortality of larger individuals, which in turn should result in higher average body mass of harvested individuals (Lindstedt & Boyce, 1985; McNab, 1971). In regions with pronounced seasonality, commonly associated with cold winters and long snow cover, fast body mass gains as newborn and juvenile are needed in order to survive prolonged periods with limited forage (Herfindal et al., 2006; Lindstedt & Boyce, 1985; Veylit et al., 2020).

Our results show that the effects of climatic conditions on trade‐offs between life‐history traits may vary in relation to habitat: chamois inhabiting mountain ranges with high proportion of forest cover, with comparatively mild winters and associated higher natural survival, may allocate the available resources more into early reproduction rather than into growth, which results in a faster pace of life with higher rates of body mass senescence (Mason et al., 2011; Veylit et al., 2020). The variation in body mass patterns observed in chamois may therefore be explained by different energy allocation between life‐history traits (Larue et al., 2021; Morin et al., 2016; Stearns, 1992). In this context, our results suggest that the resource allocation especially of young chamois in mountain ranges with a low proportion of forest cover is more focused on somatic growth and survival rather than on early reproduction (see also Hamel & Côté, 2009).

The smaller differences in growth patterns in relation to forest cover for males compared to females may be related to sex‐specific differences in habitat use and territoriality: females with kids are not territorial and generally use high‐altitude alpine meadows from spring to autumn, which allows them to exploit highly nutritious food patches (Ferrari et al., 1988). In chamois, territorial and non‐territorial males compete to monopolize groups of females during the breeding season in autumn (Corlatti, Fattorini et al., 2015), and territorial males occupy their breeding territories, which are normally located at lower elevations, as early as spring (Unterthiner et al., 2012; von Hardenberg et al., 2000). This results in a more even distribution of males across different‐quality habitats within a mountain range compared to females. Especially in mountain ranges with low proportional forest cover, this suggests that a portion of males remain in breeding territories at lower altitudes with high forest cover and lower vegetation quality also in spring and summer, which in turn could result in smaller differences in male growth patterns across and between mountain ranges. In addition, older and heavier males in polygynous species typically have higher reproductive success compared to younger and smaller males (Newbolt et al., 2017; Willisch et al., 2012). Also, males in polygynous species typically reach peak body mass and sexual maturity later than females (Zedrosser et al., 2007), including chamois (Bassano et al., 2003). Therefore, subadult male chamois likely allocate more resources into growth and less into early reproduction compared to subadult females.

Our findings are in line with Loison, Jullien et al. (1999) and Bleu et al. (2015), who suggest that the effect of spring temperatures and climate change on life‐history traits of chamois may vary in relation to the habitat. In red deer, Post and Stenseth (1999) suggest that the spatial variation of body mass in response to climatic conditions may be explained by lower sensitivity of woody plants to climatic variability in comparison to herbaceous plants, which are more likely to occur in open alpine areas. Alpine herbivores generally are capital breeders and need to maximize energy accumulation during summer (e.g., mountain goats *Oreamnos americanus*: Hamel & Côté, 2009; Alpine chamois: Apollonio et al., 2020; Morin et al., 2016). Among other things, food acquisition is limited by the available time to find resources (Toïgo et al., 2002), the need to process food, and the avoidance of abiotic constraints such as heat stress. In accordance with our results, Malagnino et al. (2021) have shown that during hot days, chamois in open landscapes spend more time resting than foraging compared to chamois in forested areas. This suggests that the variation in the effects of increasing temperatures on chamois body mass across habitats with different proportions of forest cover may be related to behavioral adaptions to increasing temperatures, such as decreases in the time spent foraging and increases in the time spent resting and relocating (*cf*. Reiner et al., 2021).

The differences in body mass between the cold and the warm subperiod changed with age in both sexes. The reasons for this as well as the associated trade‐off with body mass remain unclear, but may be related to compensatory growth (Rughetti & Festa‐Bianchet, 2010) during years with more favorable climatic conditions (Figure 3). Another explanation may be related to increased natural mortality of smaller individuals resulting in higher average body mass of harvested chamois, or alternatively, a shift of reproductive effort toward older ages (Mason et al., 2011). Larue et al. (2021) have shown in bighorn sheep and mountain goats that annual body mass gain can reveal past reproductive effort, with lower body mass gains in reproducing compared to non‐reproducing females. This suggests that chamois in mountain ranges with low forest cover may spend fewer resources on reproduction in the warm period, or that the reproductive effort has shifted toward older ages, e.g., later onset of primiparity and/or lower early birth rates accompanied by a longer lifetime reproductive period.

This study confirmed previous findings, i.e., that the effect of increasing temperatures on life‐history traits, such as body mass (Reiner et al., 2021), survival (Bleu et al., 2015), or recruitment (Chirichella et al., 2021), can differ significantly between habitats within the same species. It seems plausible that the decreasing trend of body mass in Alpine chamois will have far‐reaching impacts on populations living in open alpine habitats, since higher body mass is expected to improve winter survival for both sexes, and reproduction in females (Festa‐Bianchet et al., 2019; Stearns, 1992). Though spring temperatures are generally increasing in our study area (Figure 3a), previous research has not yet shown a decrease in average snow depth (Reiner et al., 2021), suggesting that climate‐induced declines in body mass may potentially lead to increased winter mortality due to lower energy reserves of chamois, especially at high elevations (Reiner, 2015; Rughetti et al., 2011). Whether chamois respond to increasing temperatures by moving to lower altitudes will also depend on other factors such as the presence of large predators, the availability of suitable food resources, resource competition with other ungulates (Corlatti et al., 2019), as well as on human‐wildlife conflicts in relation to forestry and recreation (*cf*. Ciach & Pęksa, 2019; Schnidrig‐Petrig & Ingold, 2001).

## CONFLICT OF INTERESTS

None.

## AUTHOR CONTRIBUTION

**Rudolf Reiner:** Conceptualization (lead); Data curation (lead); Formal analysis (lead); Investigation (lead); Methodology (equal); Project administration (lead); Resources (lead); Validation (equal); Visualization (lead); Writing – original draft (lead); Writing – review & editing (equal). **Andreas Zedrosser:** Conceptualization (supporting); Formal analysis (supporting); Investigation (supporting); Methodology (equal); Supervision (equal); Validation (equal); Writing – review & editing (equal). **Hubert Zeiler:** Conceptualization (supporting); Supervision (supporting); Validation (supporting); Writing – review & editing (supporting). **Klaus Hackländer:** Funding acquisition (lead); Project administration (supporting); Supervision (equal); Writing – review & editing (supporting). **Luca Corlatti:** Conceptualization (equal); Investigation (supporting); Methodology (equal); Supervision (equal); Validation (equal); Writing – review & editing (equal).

## Supporting information

Appendix S1

## Data Availability

The data that support the findings of this study are available at https://doi.org/10.6084/m9.figshare.19123079.v1.

## References

[ece38650-bib-0001] Akman, O. , & Whitman, D. (2008). Analysis of body size and fecundity in a grasshopper. Journal of Orthoptera Research, 17(2), 249–257. 10.1665/1082-6467-17.2.249

[ece38650-bib-0002] Albon, S. D. , & Langvatn, R. (1992). Plant phenology and the benefits of migration in a temperate ungulate. Oikos, 65(3), 502–513. 10.2307/3545568

[ece38650-bib-0127] Apollonio, M. , Merli, E. , Chirichella, R. , Pokorny, B. , Alagić, A. , Flajšman, K. , & Stephens, P. A. (2020). Capital‐income breeding in male ungulates: Causes and consequences of strategy differences among species. Frontiers in Ecology and Evolution, 8, 521767. 10.3389/fevo.2020.521767

[ece38650-bib-0003] Ascenzi, P. , Clementi, M. E. , Condò, S. G. , Coletta, M. , Petruzzelli, R. , Polizio, F. , Rizzi, M. , Giunta, C. , Peracino, V. , & Giardina, B. (1993). Functional, spectroscopic and structural properties of haemoglobin from chamois (*Rupicapra rupicapra*) and steinbock (*Capra hircus ibex*). Biochemical Journal, 296(2), 361–365. 10.1042/bj2960361 PMC1137704 8257425

[ece38650-bib-0004] Bårdsen, B.‐J. , Henden, J.‐A. , Fauchald, P. , Tveraa, T. , & Stien, A. (2011). Plastic reproductive allocation as a buffer against environmental stochasticity – linking life history and population dynamics to climate. Oikos, 120(2), 245–257. 10.1111/j.1600-0706.2010.18597.x

[ece38650-bib-0005] Bassano, B. , Perrone, A. , & von Hardenberg, A. (2003). Body weight and horn development in Alpine chamois, *Rupicapra rupicapra* (Bovidae, Caprinae). Mammalia, 67, 65–73. 10.1515/mamm.2003.67.1.65

[ece38650-bib-0006] Bates, D. , Mächler, M. , Bolker, B. , & Walker, S. (2015). Fitting linear mixed‐effects models using lme4. Journal of Statistical Software, 1(1), 67(1), 1–48. 10.18637/jss.v067.i01

[ece38650-bib-0007] Beauplet, G. , Barbraud, C. , Dabin, W. , Küssener, C. , & Guinet, C. (2006). Age‐specific survival and reproductive performances in fur seals: Evidence of senescence and individual quality. Oikos, 112(2), 430–441. 10.1111/j.0030-1299.2006.14412.x

[ece38650-bib-0008] Bergqvist, G. , Paulson, S. , & Elmhagen, B. (2018). Effects of female body mass and climate on reproduction in northern wild boar. Wildlife Biology, 2018(1). 10.2981/wlb.00421

[ece38650-bib-0009] Bischof, R. , Loe, L. E. , Meisingset, E. L. , Zimmermann, B. , Van Moorter, B. , & Mysterud, A. (2012). A migratory northern ungulate in the pursuit of spring: Jumping or surfing the green wave? The American Naturalist, 180(4), 407–424. 10.1086/667590 22976006

[ece38650-bib-0010] Blanckenhorn, W. U. (2000). The evolution of body size: What keeps organisms small? The Quarterly Review of Biology, 75(4), 385–407. 11125698 10.1086/393620

[ece38650-bib-0011] Bleu, J. , Herfindal, I. , Loison, A. , Kwak, A. M. G. , Garel, M. , Toïgo, C. , Rempfler, T. , Filli, F. , & Sæther, B.‐E. (2015). Age‐specific survival and annual variation in survival of female chamois differ between populations. Oecologia, 179(4), 1091–1098. 10.1007/s00442-015-3420-5 26290356

[ece38650-bib-0012] Bliss, L. C. (1962). Adaptations of arctic and alpine plants to environmental conditions. Arctic, 15(2), 117–144.

[ece38650-bib-0013] Bubenik, A. B. (1984). Ernährung, Verhalten und Umwelt des Schalenwildes. BLV‐Verlagsgesellschaft.

[ece38650-bib-0014] Caliński, T. , & Harabasz, J. (1974). A dendrite method for cluster analysis. Communications in Statistics, 3(1), 1–27. 10.1080/03610927408827101

[ece38650-bib-0015] Chastel, O. , Weimerskirch, H. , & Jouventin, P. (1995). Body condition and seabird reproductive performance: A study of three petrel species. Ecology, 76(7), 2240–2246. 10.2307/1941698

[ece38650-bib-0016] Chirichella, R. , Stephens, P. A. , Mason, T. H. E. , & Apollonio, M. (2021). Contrasting effects of climate change on alpine chamois. The Journal of Wildlife Management, 85, 109–120. 10.1002/jwmg.21962

[ece38650-bib-0017] Ciach, M. , & Pęksa, Ł. (2018). Impact of climate on the population dynamics of an alpine ungulate: A long‐term study of the Tatra chamois *Rupicapra rupicapra tatrica* . International Journal of Biometeorology, 62(12), 2173–2182. 10.1007/s00484-018-1619-y 30276475 PMC6244863

[ece38650-bib-0018] Ciach, M. , & Pęksa, Ł. (2019). Human‐induced environmental changes influence habitat use by an ungulate over the long term. Current Zoology, 65(2), 129–137. 10.1093/cz/zoy035 30936901 PMC6430970

[ece38650-bib-0019] Clutton‐Brock, T. H. (1988). Reproductive success: Studies of individual variation in contrasting breeding systems. University of Chicago Press.

[ece38650-bib-0020] Clutton‐Brock, T. H. , Guinness, F. E. , & Albon, S. D. (1982). Red deer: Behavior and ecology of two sexes. University of Chicago press.

[ece38650-bib-0021] Clutton‐Brock, T. H. , Stevenson, I. R. , Marrow, P. , MacColl, A. D. , Houston, A. I. , & McNamara, J. M. (1996). Population fluctuations, reproductive costs and life‐history tactics in female Soay sheep. Journal of Animal Ecology, 65(6), 675–689. 10.2307/5667

[ece38650-bib-0022] Cook, J. G. , Irwin, L. L. , Bryant, L. D. , Riggs, R. A. , & Thomas, J. W. (1998). Relations of forest cover and condition of elk: A test of the thermal cover hypothesis in summer and winter. Wildlife Monographs, 3–61.

[ece38650-bib-0023] Copernicus Land Monitoring Service . (2018). Copernicus landuse data. Copernicus Land Monitoring Service.

[ece38650-bib-0024] Corlatti, L. , Bonardi, A. , Bragalanti, N. , & Pedrotti, L. (2019). Long‐term dynamics of Alpine ungulates suggest interspecific competition. Journal of Zoology, 309(4), 241–249. 10.1111/jzo.12716

[ece38650-bib-0025] Corlatti, L. , Fattorini, L. , & Nelli, L. (2015). The use of block counts, mark‐resight and distance sampling to estimate population size of a mountain‐dwelling ungulate. Population Ecology, 57, 409–419. 10.1007/s10144-015-0481-6

[ece38650-bib-0026] Corlatti, L. , Gugiatti, A. , & Imperio, S. (2015). Horn growth patterns in Alpine chamois. Zoology, 118(3), 213–219. 10.1016/j.zool.2015.01.003 25869383

[ece38650-bib-0027] Corlatti, L. , Lorenzini, R. , & Lovari, S. (2011). The conservation of the chamois *Rupicapra* spp. Mammal Review, 41(2), 163–174. 10.1111/j.1365-2907.2011.00187.x

[ece38650-bib-0028] Davies, R. G. , Orme, C. D. L. , Olson, V. , Thomas, G. H. , Ross, S. G. , Ding, T.‐S. , Rasmussen, P. C. , Stattersfield, A. J. , Bennett, P. M. , & Blackburn, T. M. (2006). Human impacts and the global distribution of extinction risk. Proceedings of the Royal Society B: Biological Sciences, 273(1598), 2127–2133. 10.1098/rspb.2006.3551 PMC1635517 16901831

[ece38650-bib-0029] Dormann, C. , McPherson, J. , Araújo, M. , Bivand, R. , Bolliger, J. , Carl, G. , Davies, R. , Hirzel, A. , Jetz, W. , & Kissling, D. (2007). Methods to account for spatial autocorrelation in the analysis of species distributional data: A review. Ecography, 30(5), 609–628.

[ece38650-bib-0030] Douhard, F. , Gaillard, J.‐M. , Pellerin, M. , Jacob, L. , & Lemaître, J.‐F. (2017). The cost of growing large: Costs of post‐weaning growth on body mass senescence in a wild mammal. Oikos, 126(9), 1329–1338. 10.1111/oik.04421

[ece38650-bib-0031] Ericsson, G. , Ball, J. P. , & Danell, K. (2002). Body mass of moose calves along an altitudinal gradient. The Journal of Wildlife Management, 66(1), 91–97. 10.2307/3802875

[ece38650-bib-0032] ESRI Inc . (2021). ArcGIS Pro (2.8). ESRI Inc. Retrieved from https://www.esri.com/en‐us/arcgis/products/arcgis‐pro/

[ece38650-bib-0033] Ferrari, C. , Rossi, G. , & Cavani, C. (1988). Summer food habits and quality of female, kid and subadult Apennine chamois, *Rupicapra pyrenaica ornata* Neumann, 1899 (Artiodactyla, Bovidae). Zeitschrift Für Säugetierkunde, 53, 170–177.

[ece38650-bib-0034] Festa‐Bianchet, M. , Côté, S. D. , Hamel, S. , & Pelletier, F. (2019). Long‐term studies of bighorn sheep and mountain goats reveal fitness costs of reproduction. Journal of Animal Ecology, 88(8), 1118–1133. 10.1111/1365-2656.13002 31183864

[ece38650-bib-0035] Festa‐Bianchet, M. , Gaillard, J. M. , & Jorgenson, J. T. (1998). Mass‐ and density‐dependent reproductive success and reproductive costs in a capital breeder. The American Naturalist, 152(3), 367–379. 10.1086/286175 18811445

[ece38650-bib-0036] Forchhammer, M. , Clutton‐Brock, T. , Lindström, J. , & Albon, S. (2001). Climate and population density induce long‐term cohort variation in a northern ungulate. Journal of Animal Ecology, 70, 721–729. 10.1046/j.0021-8790.2001.00532.x

[ece38650-bib-0037] Gaillard, J. M. , Festa‐Bianchet, M. , Delorme, D. , & Jorgenson, J. (2000). Body mass and individual fitness in female ungulates: Bigger is not always better. Proceedings. Biological Sciences, 267(1442), 471–477. 10.1098/rspb.2000.1024 10737404 PMC1690550

[ece38650-bib-0038] Garel, M. , Loison, A. , Jullien, J.‐M. , Dubray, D. , Maillard, D. , & Gaillard, J.‐M. (2009). Sex‐specific growth in alpine chamois. Journal of Mammalogy, 90(4), 954–960.

[ece38650-bib-0039] Garel, M. , Solberg, E. J. , SÆther, B.‐E. , Herfindal, I. , & Høgda, K.‐A. (2006). The length of growing season and adult sex ratio affect sexual size dimorphism in moose. Ecology, 87(3), 745–758. 16602303 10.1890/05-0584

[ece38650-bib-0040] Geist, V. (1987). Bergmann’s rule is invalid. Canadian Journal of Zoology, 65(4), 1035–1038.

[ece38650-bib-0041] Gibbens, J. , & Arnould, J. P. Y. (2009). Age‐specific growth, survival, and population dynamics of female Australian fur seals. Canadian Journal of Zoology, 87(10), 902–911. 10.1139/Z09-080

[ece38650-bib-0042] Gosselin, J. , Zedrosser, A. , Swenson, J. E. , & Pelletier, F. (2015). The relative importance of direct and indirect effects of hunting mortality on the population dynamics of brown bears. Proceedings of the Royal Society B: Biological Sciences, 282(1798), 20141840. 10.1098/rspb.2014.1840 PMC4262167 25392469

[ece38650-bib-0043] Grassler, F. (1984). Alpenvereinseinteilung der Ostalpen (AVE). Berg’, 84, 215–224.

[ece38650-bib-0044] Green, W. C. H. (1990). Reproductive effort and associated costs in bison (Bison bison): Do older mothers try harder? Behavioral Ecology, 1(2), 148–160. 10.1093/beheco/1.2.148

[ece38650-bib-0045] Hamel, S. , & Côté, S. D. (2009). Foraging decisions in a capital breeder: Trade‐offs between mass gain and lactation. Oecologia, 161(2), 421–432. 10.1007/s00442-009-1377-y 19488787

[ece38650-bib-0046] Hamel, S. , Côté, S. D. , & Festa‐Bianchet, M. (2010). Maternal characteristics and environment affect the costs of reproduction in female mountain goats. Ecology, 91(7), 2034–2043. 10.1890/09-1311.1 20715626

[ece38650-bib-0047] Hamel, S. , Gaillard, J.‐M. , Festa‐Bianchet, M. , & CôTE, S. D. (2009). Individual quality, early‐life conditions, and reproductive success in contrasted populations of large herbivores. Ecology, 90(7), 1981–1995. 19694145 10.1890/08-0596.1

[ece38650-bib-0048] Hamel, S. , Garel, M. , Festa‐Bianchet, M. , Gaillard, J.‐M. , & Côté, S. (2009). Spring Normalized Difference Vegetation Index (NDVI) predicts annual variation in timing of peak faecal crude protein in mountain ungulates. Journal of Applied Ecology, 46, 582–589. 10.1111/j.1365-2664.2009.01643.x

[ece38650-bib-0049] Herfindal, I. , Haanes, H. , Solberg, E. J. , Røed, K. H. , Høgda, K. A. , & Sæther, B.‐E. (2014). Moose body mass variation revisited: Disentangling effects of environmental conditions and genetics. Oecologia, 174(2), 447–458. 10.1007/s00442-013-2783-8 24091427

[ece38650-bib-0050] Herfindal, I. , Solberg, E. J. , Saether, B.‐E. , Høgda, K. , & Andersen, R. (2006). Environmental phenology and geographical gradients in moose body mass. Oecologia, 150, 213–224. 10.1007/s00442-006-0519-8 16944246

[ece38650-bib-0051] Herfindal, I. , Tveraa, T. , Stien, A. , Solberg, E. J. , & Grøtan, V. (2020). When does weather synchronize life‐history traits? Spatiotemporal patterns in juvenile body mass of two ungulates. Journal of Animal Ecology, 89(6), 1419–1432. 10.1111/1365-2656.13192 32108334

[ece38650-bib-0052] Kourkgy, C. , Garel, M. , Appolinaire, J. , Loison, A. , & Toïgo, C. (2016). Onset of autumn shapes the timing of birth in Pyrenean chamois more than onset of spring. Journal of Animal Ecology, 85(2), 581–590. 10.1111/1365-2656.12463 26503480

[ece38650-bib-0053] Krüger, O. (2002). Dissecting common buzzard lifespan and lifetime reproductive success: The relative importance of food, competition, weather, habitat and individual attributes. Oecologia, 133(4), 474–482. 10.1007/s00442-002-1053-y 28466174

[ece38650-bib-0054] Larue, B. , Pelletier, F. , Côté, S. D. , Hamel, S. , & Festa‐Bianchet, M. (2021). Growth and reproduction trade‐offs can estimate previous reproductive history in alpine ungulates. Journal of Applied Ecology, 58, 869–878. 10.1111/1365-2664.13840

[ece38650-bib-0056] LeBlanc, M. , Festa‐Bianchet, M. , & Jorgenson, J. T. (2001). Sexual size dimorphism in bighorn sheep (*Ovis canadensis*): Effects of population density. Canadian Journal of Zoology, 79(9), 1661–1670. 10.1139/z01-128

[ece38650-bib-0057] Lindstedt, S. L. , & Boyce, M. S. (1985). Seasonality, fasting endurance, and body size in mammals. The American Naturalist, 125(6), 873–878.

[ece38650-bib-0058] Loison, A. , Gaillard, J.‐M. , Pélabon, C. , & Yoccoz, N. G. (1999). What factors shape sexual size dimorphism in ungulates? Evolutionary Ecology Research, 1(5), 611–633.

[ece38650-bib-0059] Loison, A. , Jullien, J.‐M. , & Menaut, P. (1999). Relationship between Chamois and Isard survival and variation in global and local climate regimes: Contrasting examples from the alps and Pyrenees. Ecological Bulletins, 47, 126–136.

[ece38650-bib-0060] Loison, A. , Langvatn, R. , & Solberg, E. J. (1999). Body mass and winter mortality in red deer calves: Disentangling sex and climate effects. Ecography, 22, 20–30. 10.1111/j.1600-0587.1999.tb00451.x

[ece38650-bib-0061] Mainguy, J. , & Côté, S. D. (2008). Age‐ and state‐dependent reproductive effort in male mountain goats, *Oreamnos americanus* . Behavioral Ecology and Sociobiology, 62(6), 935–943.

[ece38650-bib-0062] Malagnino, A. , Börger, L. , Courbin, N. , Bonnot, N. , Marchand, P. , Morellet, N. , & Loison, A. (2021). Summer activity budgets of chamois in relation to heat, food and disturbance constraints. International Rupicapra Symposium, III, 54–55.

[ece38650-bib-0063] Mason, T. , Apollonio, M. , Chirichella, R. , Willis, S. , & Stephens, P. (2014). Environmental change and long‐term body mass declines in an alpine mammal. Frontiers in Zoology, 11(69), 69. 10.1186/s12983-014-0069-6

[ece38650-bib-0064] Mason, T. , Chirichella, R. , Richards, S. , Stephens, P. , Willis, S. , & Apollonio, M. (2011). Contrasting life histories in neighbouring populations of a large mammal. PLoS One, 6, e28002. 10.1371/journal.pone.0028002 22125651 PMC3220718

[ece38650-bib-0065] Mautz, W. W. (1978). Sledding on a bushy hillside: The fat cycle in deer. Wildlife Society Bulletin (1973‐2006), 6(2), 88–90.

[ece38650-bib-0066] McLellan, B. N. (2011). Implications of a high‐energy and low‐protein diet on the body composition, fitness, and competitive abilities of black (*Ursus americanus*) and grizzly (*Ursus arctos*) bears. Canadian Journal of Zoology, 89(6), 546–558.

[ece38650-bib-0067] McLoughlin, P. D. , Gaillard, J.‐M. , Boyce, M. S. , Bonenfant, C. , Messier, F. , Duncan, P. , Delorme, D. , Moorter, B. V. , Saïd, S. , & Klein, F. (2007). Lifetime reproductive success and composition of the home range in a large herbivore. Ecology, 88(12), 3192–3201. 10.1890/06-1974.1 18229853

[ece38650-bib-0068] McNab, B. K. (1971). On the ecological significance of Bergmann’s rule. Ecology, 52(5), 845–854. 10.2307/1936032

[ece38650-bib-0069] Merkle, J. A. , Monteith, K. L. , Aikens, E. O. , Hayes, M. M. , Hersey, K. R. , Middleton, A. D. , Oates, B. A. , Sawyer, H. , Scurlock, B. M. , & Kauffman, M. J. (2016). Large herbivores surf waves of green‐up during spring. Proceedings. Biological Sciences, 283(1833), 20160456. 10.1098/rspb.2016.0456 27335416 PMC4936031

[ece38650-bib-0070] Middleton, A. , Merkle, J. , McWhirter, D. , Cook, J. , Cook, R. , White, P. , & Kauffman, M. (2018). Green‐wave surfing increases fat gain in a migratory ungulate. Oikos, 127, 1060–1068. 10.1111/oik.05227

[ece38650-bib-0071] Milligan, G. W. , & Cooper, M. C. (1985). An examination of procedures for determining the number of clusters in a data set. Psychometrika, 50(2), 159–179. 10.1007/BF02294245

[ece38650-bib-0072] Morin, A. , Rughetti, M. , Rioux‐Paquette, S. , & Festa‐Bianchet, M. (2016). Older conservatives: Reproduction in female alpine chamois (*Rupicapra rupicapra*) is increasingly risk‐averse with age. Canadian Journal of Zoology, 94(5), 311–321. 10.1139/cjz-2015-0153

[ece38650-bib-0073] Mysterud, A. , Langvatn, R. , Yoccoz, N. G. , & Stenseth, N. C. (2001). Plant phenology, migration and geographical variation in body weight of a large herbivore: The effect of a variable topography. Journal of Animal Ecology, 70(6), 915–923. http://www.jstor.org/stable/2693495

[ece38650-bib-0074] Mysterud, A. , Langvatn, R. , Yoccoz, N. G. , & Stenseth, N. C. H. R. (2002). Large‐scale habitat variability, delayed density effects and red deer populations in Norway. Journal of Animal Ecology, 71(4), 569–580.

[ece38650-bib-0075] Newbolt, C. H. , Acker, P. K. , Neuman, T. J. , Hoffman, S. I. , Ditchkoff, S. S. , & Steury, T. D. (2017). Factors influencing reproductive success in male white‐tailed deer. The Journal of Wildlife Management, 81(2), 206–217. 10.1002/jwmg.21191

[ece38650-bib-0076] Ozgul, A. , Childs, D. Z. , Oli, M. K. , Armitage, K. B. , Blumstein, D. T. , Olson, L. E. , Tuljapurkar, S. , & Coulson, T. (2010). Coupled dynamics of body mass and population growth in response to environmental change. Nature, 466, 482–485. 10.1038/nature09210 20651690 PMC5677226

[ece38650-bib-0077] Parker, K. L. , Barboza, P. S. , & Gillingham, M. P. (2009). Nutrition integrates environmental responses of ungulates. Functional Ecology, 23(1), 57–69. 10.1111/j.1365-2435.2009.01528.x

[ece38650-bib-0078] Pelletier, F. , Réale, D. , Garant, D. , Coltman, D. W. , & Festa‐Bianchet, M. (2007). Selection on heritable seasonal phenotypic plasticity of body mass. Evolution; International Journal of Organic Evolution, 61(8), 1969–1979. 10.1111/j.1558-5646.2007.00160.x 17683438

[ece38650-bib-0079] Pérez‐Barbería, F. J. , García, A. J. , Cappelli, J. , Landete‐Castillejos, T. , Serrano, M. P. , & Gallego, L. (2020). Heat stress reduces growth rate of red deer calf: Climate warming implications. PLoS One, 15(6), e0233809. 10.1371/journal.pone.0233809 32480402 PMC7263848

[ece38650-bib-0080] Perez‐Barberia, F. J. , Robles, L. , & Nores, C. (1996). Horn growth pattern in Cantabrian chamois *Rupicapra pyrenaica parva*: Influence of sex, location and phaenology. Acta Theriologica, 41, 83–92.

[ece38650-bib-0081] Pettorelli, N. , Dray, S. , Gaillard, J.‐M. , Chessel, D. , Duncan, P. , Illius, A. , Guillon, N. , Klein, F. , & Van Laere, G. (2003). Spatial variation in springtime food resources influences the winter body mass of roe deer fawns. Oecologia, 137(3), 363–369. 10.1007/s00442-003-1364-7 12920639

[ece38650-bib-0082] Pettorelli, N. , Gaillard, J.‐M. , Duncan, P. , Ouellet, J.‐P. , & Van Laere, G. (2001). Population density and small‐scale variation in habitat quality affect phenotypic quality in roe deer. Oecologia, 128(3), 400–405. 24549909 10.1007/s004420100682

[ece38650-bib-0083] Plard, F. , Gaillard, J.‐M. , Coulson, T. , Hewison, A. J. M. , Douhard, M. , Klein, F. , Delorme, D. , Warnant, C. , & Bonenfant, C. (2015). The influence of birth date via body mass on individual fitness in a long‐lived mammal. Ecology, 96(6), 1516–1528. 10.1890/14-0106.1

[ece38650-bib-0084] Polák, J. , & Frynta, D. (2009). Sexual size dimorphism in domestic goats, sheep, and their wild relatives. Biological Journal of the Linnean Society, 98(4), 872–883. 10.1111/j.1095-8312.2009.01294.x

[ece38650-bib-0085] Post, E. , & Stenseth, N. C. (1999). Climatic variability, plant phenology, and northern ungulates. Ecology, 80(4), 1322–1339.

[ece38650-bib-0086] R Core Team (2020). R: A language and environment for statistical computing. R Foundation for Statistical Computing. Retrieved from https://www.r‐project.org/

[ece38650-bib-0087] Reiner, R. (2015). Populationsdynamik und Bestandestrends beim Gamswild (*Rupicapra rupicapra*): Untersuchung, Darstellung und Faktorenanalyse für das Bundesland Salzburg. University of Natural Resources and Life Sciences.

[ece38650-bib-0088] Reiner, R. , Zedrosser, A. , Zeiler, H. , Hackländer, K. , & Corlatti, L. (2020). Population reconstruction as an informative tool for monitoring chamois populations. Wildlife Biology, 2020(4). 10.2981/wlb.00757

[ece38650-bib-0089] Reiner, R. , Zedrosser, A. , Zeiler, H. , Hackländer, K. , & Corlatti, L. (2021). Forests buffer the climate‐induced decline of body mass in a mountain herbivore. Global Change Biology, 27, 3741–3752. 10.1111/gcb.15711 33993622 PMC8361913

[ece38650-bib-0090] Ritchot, Y. , Festa‐Bianchet, M. , Coltman, D. , & Pelletier, F. (2021). Determinants and long‐term costs of early reproduction in males of a long‐lived polygynous mammal. Ecology and Evolution, 11, 6829–6845. 10.1002/ece3.7530 34141259 PMC8207375

[ece38650-bib-0091] Rode, K. D. , Amstrup, S. C. , & Regehr, E. V. (2010). Reduced body size and cub recruitment in polar bears associated with sea ice decline. Ecological Applications: A Publication of the Ecological Society of America, 20(3), 768–782. 10.1890/08-1036.1 20437962

[ece38650-bib-0092] Ronget, V. , Gaillard, J.‐M. , Coulson, T. , Garratt, M. , Gueyffier, F. , Lega, J.‐C. , & Lemaître, J.‐F. (2018). Causes and consequences of variation in offspring body mass: Meta‐analyses in birds and mammals. Biological Reviews, 93(1), 1–27. 10.1111/brv.12329 28393457

[ece38650-bib-0093] RStudio Team (2019). RStudio: Integrated development for R. RStudio Inc.

[ece38650-bib-0094] Rughetti, M. , & Festa‐Bianchet, M. (2010). Compensatory growth limits opportunities for artificial selection in alpine chamois. The Journal of Wildlife Management, 74(5), 1024–1029. 10.2193/2009-335

[ece38650-bib-0095] Rughetti, M. , & Festa‐Bianchet, M. (2011). Seasonal changes in sexual size dimorphism in northern chamois. Journal of Zoology, 284(4), 257–264. 10.1111/j.1469-7998.2011.00800.x

[ece38650-bib-0096] Rughetti, M. , & Festa‐Bianchet, M. (2012). Effects of spring–summer temperature on body mass of chamois. Journal of Mammalogy, 93, 1301–1307. 10.1644/11-MAMM-A-402.1

[ece38650-bib-0097] Rughetti, M. , Toïgo, C. , Von Hardenberg, A. , Rocchia, E. , & Festa‐Bianchet, M. (2011). Effects of an exceptionally snowy winter on chamois survival. Acta Theriologica, 56(4), 329–333. 10.1007/s13364-011-0040-2

[ece38650-bib-0098] Sæther, B.‐E. , Andersen, R. , Hjeljord, O. , & Heim, M. (1996). Ecological correlates of regional variation in life history of the moose *Alces alces* . Ecology, 77(5), 1493–1500. 10.2307/2265546

[ece38650-bib-0099] Sand, H. , Cederlund, G. , & Danell, K. (1995). Geographical and latitudinal variation in growth patterns and adult body size of Swedish moose (*Alces alces*). Oecologia, 102(4), 433–442. 28306886 10.1007/BF00341355

[ece38650-bib-0100] Schnidrig‐Petrig, R. , & Ingold, P. (2001). Effects of paragliding on alpine chamois *Rupicapra rupicapra rupicapra* . Wildlife Biology, 7(3), 285–294. 10.2981/wlb.2001.033

[ece38650-bib-0101] Schröder, W. , & von Elsner‐Schack, I. (1985). Correct age determination in chamois. In S. Lovari (Ed.), The biology and management of mountain ungulates (pp. 65–70). Croom Helm.

[ece38650-bib-0102] Sparling, C. E. , Speakman, J. R. , & Fedak, M. A. (2006). Seasonal variation in the metabolic rate and body composition of female grey seals: Fat conservation prior to high‐cost reproduction in a capital breeder? Journal of Comparative Physiology B, 176(6), 505–512. 10.1007/s00360-006-0072-0 16506041

[ece38650-bib-0103] Stearns, S. (1989). Trade‐offs in life‐history evolution. Functional Ecology, 3(3), 259–268.

[ece38650-bib-0104] Stearns, S. (Ed.) (1992). The evolution of life histories. Oxford University Press.

[ece38650-bib-0105] Sturm, R. (2016). Relationship between body size and reproductive capacity in females of the black field cricket (Orthoptera, Gryllidae). Linzer Biologische Beiträge, 48, 1823–1834.

[ece38650-bib-0106] Swenson, J. E. , Adamič, M. , Huber, D. , & Stokke, S. (2007). Brown bear body mass and growth in northern and southern Europe. Oecologia, 153(1), 37–47. 10.1007/s00442-007-0715-1 17415593

[ece38650-bib-0107] Toïgo, C. , Gaillard, J.‐M. , Festa‐Bianchet, M. , Largo, E. , Michallet, J. , & Maillard, D. (2007). Sex‐ and age‐specific survival of the highly dimorphic Alpine ibex: Evidence for a conservative life‐history tactic. Journal of Animal Ecology, 76(4), 679–686. 10.1111/j.1365-2656.2007.01254.x 17584373

[ece38650-bib-0108] Toïgo, C. , Gaillard, J.‐M. , Gauthier, D. , Girard, I. , Martinot, J.‐P. , & Michallet, J. (2002). Female reproductive success and costs in an alpine capital breeder under contrasting environments. Écoscience, 9(4), 427–433. 10.1080/11956860.2002.11682730

[ece38650-bib-0109] Toïgo, C. , Gaillard, J.‐M. , & Loison, A. (2013). Alpine ibex males grow large horns at no survival cost for most of their lifetime. Oecologia, 173(4), 1261–1269. 10.1007/s00442-013-2700-1 23774947

[ece38650-bib-0110] Toïgo, C. , Gaillard, J. M. , Van Laere, G. , Hewison, M. , & Morellet, N. (2006). How does environmental variation influence body mass, body size, and body condition? Roe deer as a case study. Ecography, 29, 301–308. 10.1111/j.2006.0906-7590.04394.x

[ece38650-bib-0111] Tveraa, T. , Stien, A. , Bårdsen, B.‐J. , & Fauchald, P. (2013). Population densities, vegetation green‐up, and plant productivity: Impacts on reproductive success and juvenile body mass in reindeer. PLoS One, 8(2), e56450. 10.1371/journal.pone.0056450 23451049 PMC3579868

[ece38650-bib-0112] Unterthiner, S. , Ferretti, F. , Rossi, L. , & Lovari, S. (2012). Sexual and seasonal differences of space use in Alpine chamois. Ethology Ecology & Evolution, 24(3), 257–274. 10.1080/03949370.2012.658872

[ece38650-bib-0113] Van Soest, P. J. (1994). Nutritional ecology of the ruminant. Cornell University Press. 10.7591/9781501732355

[ece38650-bib-0114] Veylit, L. , Sæther, B.‐E. , Gaillard, J.‐M. , Baubet, E. , & Gamelon, M. (2020). Grow fast at no cost: No evidence for a mortality cost for fast early‐life growth in a hunted wild boar population. Oecologia, 192(4), 999–1012. 10.1007/s00442-020-04633-9 32242324 PMC7165149

[ece38650-bib-0115] von Hardenberg, A. , Bassano, B. , Peracino, A. , & Lovari, S. (2000). Male Alpine chamois occupy territories at hotspots before the mating season. Ethology, 106, 617–630. 10.1046/j.1439-0310.2000.00579.x

[ece38650-bib-0116] Wickham, H. (2016). ggplot2: Elegant graphics for data analysis. Springer‐Verlag. Retrieved from https://ggplot2.tidyverse.org

[ece38650-bib-0117] Wilder, S. M. , Raubenheimer, D. , & Simpson, S. J. (2016). Moving beyond body condition indices as an estimate of fitness in ecological and evolutionary studies. Functional Ecology, 30(1), 108–115. 10.1111/1365-2435.12460

[ece38650-bib-0118] Willisch, C. S. , Biebach, I. , Koller, U. , Bucher, T. , Marreros, N. , Ryser‐Degiorgis, M.‐P. , Keller, L. F. , & Neuhaus, P. (2012). Male reproductive pattern in a polygynous ungulate with a slow life‐history: The role of age, social status and alternative mating tactics. Evolutionary Ecology, 26(1), 187–206. 10.1007/s10682-011-9486-6

[ece38650-bib-0119] Willisch, C. , Bieri, K. , Struch, M. , Franceschina, R. , Schnidrig‐Petrig, R. , & Ingold, P. (2013). Climate effects on demographic parameters in an unhunted population of Alpine chamois (*Rupicapra rupicapra*). Journal of Mammalogy, 94, 173–182. 10.1644/10-mamm-a-278.1

[ece38650-bib-0120] Yoccoz, N. G. , Mysterud, A. , Langvatn, R. , & Stenseth, N. C. (2002). Age‐ and density‐dependent reproductive effort in male red deer. Proceedings. Biological Sciences, 269(1500), 1523–1528. 10.1098/rspb.2002.2047 12184820 PMC1691067

[ece38650-bib-0121] Yom‐Tov, Y. (2001). Global warming and body mass decline in Israeli passerine birds. Proceedings. Biological Sciences, 268(1470), 947–952. 10.1098/rspb.2001.1592 11370968 PMC1088692

[ece38650-bib-0122] Zedrosser, A. , Bellemain, E. , Taberlet, P. , & Swenson, J. E. (2007). Genetic estimates of annual reproductive success in male brown bears: The effects of body size, age, internal relatedness and population density. Journal of Animal Ecology, 76(2), 368–375. 10.1111/j.1365-2656.2006.01203.x 17302844

[ece38650-bib-0123] Zuur, A. , Ieno, E. N. , Walker, N. , Saveliev, A. A. , & Smith, G. M. (2009). Mixed effects models and extensions in ecology with R. Springer Science & Business Media.

