# Supplementary material for: Habitat and climate shape growth patterns in a mountain ungulate
Source: Ecol Evol. 2022 Mar 7;12(3):e8650. doi: 10.1002/ece3.8650 (PMC8901871; doi:10.1002/ece3.8650)
Supplement: Supplementary file 1 — Appendix S1 [file ECE3-12-e8650-s001.docx]

**Supplementary Material**

Habitat and climate shape growth patterns in a mountain ungulate

Ecology and Evolution

Reiner, R., Zedrosser, A., Zeiler, H., Hackländer, K., Corlatti, L.

**Table S1:** Names, metrics, and chamois harvest data of the mountain ranges of Salzburg, Styria and Carinthia (Austria), St. Gall Canton (Switzerland), Berchtesgaden National Park (Germany), and Liechtenstein.

| Mountain range ID | Name | Total suitable habitat | Forest cover | Number of chamois (≥ 1 year) | | Mean body mass | |
| --- | --- | --- | --- | --- | --- | --- | --- |
|  |  | (ha) | (%) | Males, *n* | Females, n | Males, kg | Females, kg |
| 9 | Lofer and Leogang Mountains | 12,817 | 77 | 1,029 | 839 | 22.5 | 18.8 |
| 10 | Berchtesgaden Alps (Austria & Germany) | 71,157 | 64 | 5,489 | 5,469 | 22.4 | 18.7 |
| 11 | Chiemgau Alps | 10,262 | 85 | 383 | 344 | 22.3 | 18.8 |
| 12 | Salzburger Schieferalpen | 37,478 | 87 | 1,109 | 883 | 21.9 | 19.4 |
| 13 | Tennen Mountain | 29,714 | 70 | 1,353 | 1,225 | 24.2 | 18.9 |
| 14 | Dachstein Mountains | 44,340 | 80 | 3,106 | 3,015 | 22.7 | 19.3 |
| 15 | Totes Gebirge | 50,087 | 77 | 2,546 | 2,356 | 22.8 | 19.5 |
| 16 | Ennstal Alps | 108,703 | 85 | 4,968 | 4,202 | 22.3 | 18.6 |
| 17a | Salzkammergut Mountains | 69,401 | 92 | 3,543 | 3,290 | 23.1 | 18.9 |
| 18 | Hochschwab | 93,708 | 85 | 7,871 | 7,092 | 22.4 | 19.1 |
| 19 | Mürzsteg Alps | 38,285 | 93 | 3,116 | 2,846 | 22.9 | 19.5 |
| 20 | Rax and Schneeberg | 6,130 | 91 | 570 | 565 | 22.9 | 18.3 |
| 21 | Ybbstal Alps | 31,488 | 96 | 2,941 | 2,443 | 21.6 | 17.8 |
| 25 | Raetikon (Liechtenstein) | 9,540 | 67 | 1,250 | 1,258 | 20.8 | 17.4 |
| 34 | Kitzbühel Alps | 37,975 | 60 | 2,709 | 2,817 | 21.8 | 19.1 |
| 35 | Zillertal Alps | 11,444 | 28 | 505 | 409 | 23.8 | 21.2 |
| 36 | Venediger Group | 26,722 | 24 | 930 | 1,033 | 24.1 | 20.3 |
| 39 | Granatspitz Group | 16,760 | 34 | 574 | 422 | 22.9 | 19.2 |
| 40 | Glockner Group | 40,318 | 27 | 2,264 | 2,354 | 23.6 | 20.1 |
| 42 | Goldberg Group | 35,698 | 40 | 1,610 | 1,815 | 23.5 | 20.2 |
| 44 | Ankogel Group | 43,271 | 47 | 2,092 | 2,617 | 22.5 | 19.1 |
| 45a | Radstadt Tauern | 63,185 | 61 | 3,077 | 3,239 | 22.5 | 19.3 |
| 45b | Schladming Tauern | 89,686 | 63 | 8,173 | 7,556 | 22.1 | 18.8 |
| 45c | Rottenmann and Wölz Tauern | 95,460 | 73 | 4,960 | 4,824 | 22.0 | 19.6 |
| 45d | Seckau Tauern | 45,736 | 83 | 2,625 | 2,613 | 22.4 | 19.4 |
| 46a | Gurktal Alps | 39,082 | 89 | 912 | 1,203 | 21.0 | 18.7 |
| 46b | Lavanttal Alps | 154,322 | 95 | 6,824 | 7,205 | 20.9 | 17.8 |
| 47 | Prealps East of the Mur | 75,404 | 99 | 2,051 | 1,886 | 21.4 | 18.4 |
| 56 | Gailtal Alps | 12,456 | 95 | 1,260 | 1,284 | 19.8 | 17.4 |
| 88 | Appenzell Alps (Swiss) | 36,361 | 54 | 6,192 | 5,631 | 22.1 | 19.0 |
| 99 | Glarn Alps (Swiss) | 53,082 | 77 | 4,921 | 5,200 | 23.8 | 20.5 |

**Table S2:** Total and annual number of chamois harvested in the mountain ranges of Salzburg, Styria and Carinthia (Austria), St. Gall Canton (Switzerland), Berchtesgaden National Park (Germany) and Liechtenstein, 1993-2109. Shaded cells indicate years where no data have been reported.

| Mountain range ID | Total | 1993 | 1994 | 1995 | 1996 | 1997 | 1998 | 1999 | 2000 | 2001 | 2002 | 2003 | 2004 | 2005 | 2006 | 2007 | 2008 | 2009 | 2010 | 2011 | 2012 | 2013 | 2014 | 2015 | 2016 | 2017 | 2018 | 2019 |
| --- | --- | --- | --- | --- | --- | --- | --- | --- | --- | --- | --- | --- | --- | --- | --- | --- | --- | --- | --- | --- | --- | --- | --- | --- | --- | --- | --- | --- |
| 9 | 1,868 |  |  |  |  |  | 98 | 81 | 69 | 83 | 85 | 76 | 81 | 75 | 70 | 74 | 79 | 83 | 85 | 79 | 90 | 94 | 80 | 103 | 93 | 102 | 93 | 95 |
| 10 | 10,958 | 203 | 310 | 270 | 216 | 195 | 557 | 448 | 430 | 467 | 506 | 504 | 476 | 460 | 415 | 444 | 478 | 457 | 427 | 504 | 404 | 349 | 367 | 409 | 427 | 457 | 449 | 329 |
| 11 | 727 |  |  |  |  |  | 38 | 32 | 31 | 26 | 28 | 31 | 29 | 27 | 24 | 25 | 38 | 31 | 34 | 31 | 37 | 36 | 34 | 48 | 37 | 40 | 43 | 27 |
| 12 | 1,992 | 9 | 8 | 7 | 4 | 6 | 82 | 80 | 63 | 59 | 89 | 74 | 82 | 96 | 87 | 72 | 79 | 84 | 99 | 102 | 94 | 85 | 105 | 111 | 113 | 98 | 107 | 97 |
| 13 | 2,578 |  |  |  |  |  | 144 | 131 | 137 | 135 | 153 | 150 | 160 | 147 | 128 | 109 | 108 | 97 | 77 | 88 | 86 | 81 | 96 | 101 | 114 | 123 | 107 | 106 |
| 14 | 6,121 | 237 | 267 | 235 | 243 | 242 | 277 | 271 | 273 | 269 | 281 | 287 | 284 | 232 | 231 | 230 | 258 | 235 | 203 | 215 | 196 | 179 | 177 | 167 | 161 | 159 | 154 | 158 |
| 15 | 4,902 | 220 | 236 | 186 | 241 | 242 | 219 | 194 | 206 | 180 | 210 | 214 | 191 | 165 | 186 | 175 | 203 | 172 | 183 | 192 | 150 | 142 | 148 | 147 | 132 | 132 | 115 | 121 |
| 16 | 9,170 | 578 | 569 | 499 | 520 | 515 | 518 | 443 | 443 | 458 | 458 | 396 | 347 | 291 | 251 | 220 | 271 | 251 | 261 | 261 | 199 | 222 | 195 | 202 | 200 | 198 | 227 | 177 |
| 17a | 6,833 | 204 | 312 | 270 | 219 | 197 | 200 | 135 | 120 | 121 | 152 | 132 | 129 | 139 | 133 | 161 | 164 | 138 | 109 | 154 | 93 | 80 | 80 | 107 | 125 | 166 | 136 | 70 |
| 18 | 14,963 | 832 | 824 | 774 | 728 | 738 | 804 | 728 | 757 | 765 | 859 | 827 | 740 | 561 | 462 | 403 | 485 | 318 | 374 | 410 | 265 | 319 | 313 | 318 | 336 | 330 | 357 | 336 |
| 19 | 5,962 | 292 | 324 | 284 | 314 | 281 | 313 | 289 | 273 | 289 | 305 | 310 | 303 | 200 | 209 | 200 | 241 | 177 | 172 | 159 | 139 | 109 | 110 | 119 | 112 | 141 | 154 | 143 |
| 20 | 1,135 | 59 | 65 | 58 | 62 | 56 | 53 | 49 | 41 | 48 | 55 | 43 | 48 | 34 | 26 | 31 | 44 | 28 | 32 | 37 | 29 | 33 | 29 | 23 | 34 | 42 | 39 | 37 |
| 21 | 5,384 | 237 | 229 | 231 | 236 | 242 | 258 | 222 | 258 | 331 | 395 | 364 | 327 | 241 | 158 | 162 | 173 | 157 | 181 | 173 | 127 | 102 | 79 | 86 | 107 | 105 | 110 | 93 |
| 25 | 2,896 | 89 | 110 | 112 | 95 | 95 | 83 | 66 | 79 | 81 | 76 | 85 | 84 | 79 | 73 | 68 | 73 | 74 | 84 | 109 | 89 | 124 | 123 | 112 | 118 | 98 | 106 | 123 |
| 34 | 5,526 | 22 | 27 | 25 | 67 | 66 | 246 | 228 | 231 | 202 | 220 | 262 | 234 | 234 | 224 | 203 | 203 | 226 | 236 | 310 | 295 | 264 | 262 | 257 | 220 | 258 | 276 | 228 |
| 35 | 914 |  |  |  |  |  | 55 | 45 | 42 | 40 | 46 | 46 | 40 | 43 | 41 | 34 | 33 | 25 | 30 | 35 | 25 | 24 | 41 | 56 | 61 | 58 | 57 | 37 |
| 36 | 1,963 |  |  |  |  |  | 145 | 144 | 143 | 132 | 134 | 116 | 82 | 52 | 63 | 54 | 75 | 70 | 63 | 84 | 75 | 61 | 81 | 97 | 68 | 84 | 75 | 65 |
| 39 | 996 |  |  |  |  |  | 64 | 71 | 74 | 69 | 77 | 72 | 58 | 55 | 28 | 32 | 35 | 29 | 24 | 34 | 29 | 27 | 37 | 39 | 34 | 45 | 36 | 27 |
| 40 | 4,618 |  |  |  |  |  | 307 | 248 | 253 | 231 | 238 | 221 | 236 | 207 | 202 | 173 | 196 | 203 | 194 | 213 | 192 | 178 | 213 | 221 | 180 | 164 | 195 | 153 |
| 42 | 3,425 |  |  |  |  |  | 157 | 150 | 167 | 160 | 174 | 171 | 160 | 174 | 192 | 164 | 160 | 183 | 137 | 160 | 132 | 124 | 159 | 152 | 138 | 156 | 134 | 121 |
| 44 | 4,709 |  |  |  |  |  | 268 | 259 | 272 | 277 | 260 | 252 | 228 | 229 | 235 | 231 | 251 | 217 | 211 | 209 | 187 | 156 | 169 | 178 | 156 | 153 | 170 | 141 |
| 45a | 6,316 |  |  |  |  |  | 301 | 330 | 333 | 328 | 319 | 329 | 323 | 337 | 312 | 265 | 307 | 289 | 255 | 281 | 257 | 245 | 240 | 286 | 260 | 257 | 277 | 185 |
| 45b | 15,729 | 427 | 468 | 440 | 447 | 462 | 798 | 743 | 804 | 778 | 789 | 763 | 717 | 673 | 737 | 659 | 654 | 610 | 575 | 595 | 523 | 492 | 474 | 481 | 429 | 400 | 408 | 383 |
| 45c | 9,784 | 496 | 503 | 458 | 529 | 532 | 489 | 472 | 542 | 445 | 448 | 437 | 405 | 364 | 359 | 318 | 325 | 322 | 314 | 333 | 282 | 254 | 208 | 227 | 171 | 176 | 190 | 185 |
| 45d | 5,238 | 258 | 271 | 257 | 267 | 288 | 298 | 286 | 298 | 274 | 285 | 275 | 200 | 198 | 189 | 168 | 177 | 139 | 132 | 114 | 114 | 101 | 112 | 113 | 101 | 109 | 120 | 94 |
| 46a | 2,115 | 35 | 35 | 37 | 48 | 53 | 94 | 91 | 123 | 132 | 130 | 116 | 109 | 88 | 90 | 85 | 88 | 95 | 79 | 100 | 77 | 71 | 62 | 65 | 58 | 54 | 57 | 43 |
| 46b | 14,029 | 401 | 388 | 387 | 403 | 403 | 426 | 462 | 469 | 468 | 473 | 526 | 517 | 545 | 546 | 554 | 553 | 563 | 535 | 561 | 583 | 545 | 560 | 530 | 622 | 654 | 675 | 680 |
| 47 | 3,937 | 170 | 130 | 138 | 135 | 124 | 119 | 130 | 126 | 139 | 147 | 151 | 148 | 148 | 132 | 151 | 139 | 135 | 144 | 155 | 162 | 165 | 135 | 139 | 143 | 166 | 190 | 176 |
| 56 | 2,566 | 22 | 27 | 25 | 67 | 66 | 83 | 76 | 80 | 86 | 86 | 114 | 95 | 107 | 96 | 103 | 66 | 106 | 116 | 144 | 175 | 133 | 125 | 117 | 79 | 116 | 123 | 111 |
| 88 | 11,823 |  |  |  | 722 | 763 | 736 | 590 | 676 | 718 | 717 | 642 | 585 | 532 | 569 | 516 | 459 | 412 | 422 | 454 | 428 | 386 | 251 | 218 | 267 | 276 | 266 | 218 |
| 99 | 10,121 |  |  |  | 592 | 649 | 688 | 442 | 448 | 526 | 593 | 524 | 451 | 460 | 395 | 387 | 356 | 397 | 393 | 431 | 391 | 373 | 296 | 264 | 270 | 277 | 264 | 254 |

**Table S3:** Sex and age specifics body mass, and chamois harvest data of the mountain ranges of Salzburg, Styria and Carinthia (Austria), St. Gall Canton (Switzerland), Berchtesgaden National Park (Germany), and Liechtenstein for two climatic subperiods.

| Age | Number of chamois  females | | Number of chamois  females | | Mean body mass males, kg | | Mean body mass females, kg | |
| --- | --- | --- | --- | --- | --- | --- | --- | --- |
|  | cold period (1993-2006) | warm period (2007-2019) | cold period (1993-2006) | warm period (2007-2019) | cold period (1993-2006) | warm period (2007-2019) | cold period (1993-2006) | warm period (2007-2019) |
| 1.5 | 10,302 | 8,710 | 9,291 | 8,153 | 15.3 | 15.0 | 14.7 | 14.4 |
| 2.5 | 6,959 | 4,703 | 6,871 | 5,191 | 19.2 | 18.9 | 17.8 | 17.2 |
| 3.5 | 4,590 | 2,598 | 6,068 | 3,768 | 21.5 | 20.9 | 18.7 | 18.0 |
| 4.5 | 3,302 | 1,443 | 4,767 | 2,646 | 22.7 | 22.4 | 19.2 | 18.7 |
| 5.5 | 2,265 | 1,079 | 3,533 | 2,236 | 23.1 | 23.2 | 19.2 | 19.1 |
| 6.5 | 2,112 | 973 | 3,047 | 1,872 | 23.6 | 23.8 | 19.5 | 19.4 |
| 7.5 | 2,044 | 1,003 | 4,103 | 2,323 | 23.9 | 24.4 | 19.7 | 19.7 |
| 8.5 | 2,038 | 907 | 3,285 | 2,208 | 24.0 | 24.6 | 20.0 | 19.9 |
| 9.5 | 1,927 | 768 | 2,670 | 1,749 | 23.9 | 24.5 | 19.7 | 19.7 |
| 10.5 | 3,065 | 997 | 2,214 | 1,263 | 23.5 | 24.2 | 19.8 | 20.3 |
| 11.5 | 2,410 | 1,069 | 1,466 | 700 | 23.5 | 24.1 | 19.9 | 19.9 |
| 12.5 | 2,293 | 847 | 1,048 | 476 | 23.1 | 23.7 | 19.7 | 19.9 |
| 13.5 | 2,003 | 590 | 628 | 238 | 23.0 | 23.1 | 19.4 | 19.7 |
| 14.5 | 1,530 | 335 | 418 | 103 | 22.6 | 22.8 | 19.4 | 19.3 |
| 15.5 | 1,186 | 193 | 258 | 45 | 22.3 | 22.3 | 19.2 | 19.7 |

**Table S4:** Highest Calinski-Harabasz (CH) index of all possible chronological groups of
spring-summer temperatures for a fixed number of climatic subperiods in mountain ranges of Salzburg. Styria and Carinthia (Austria). St. Gall Canton (Switzerland). Berchtesgaden National Park (Germany) and Liechtenstein for 1993 to 2019 and 1998 to 2019. Higher values of the CH index represent higher between-cluster variance relative to within-cluster variance. Therefore. the most probable number of climatic subperiods with different spring-summer temperatures is 2 in each case.

| Number of subperiods | Top CH index 1993-2019 | Top CH index 1998-2019 |
| --- | --- | --- |
| 2 | 13.592 | 11.042 |
| 3 | 13.045 | 10.542 |
| 4 | 12.235 | 10.011 |
| 5 | 11.312 | 10.803 |

**Table S5:** Model fitted to explain temporal variation in chamois body mass growth in mountain ranges of Salzburg. Styria and Carinthia (Austria). St. Gall Canton (Switzerland). Berchtesgaden National Park (Germany) and Liechtenstein. between 1998 and 2019. The table reports variables used in the analysis. beta estimates. standard errors (SE). t-score (t). upper and lower 95% confidence interval (CI) and *p*-value.

| **Variable** | ***Estimate*** | ***SE*** | ***t*** | ***CI_0.025_*** | ***CI_0.975_*** | ***p*** |
| --- | --- | --- | --- | --- | --- | --- |
| **females** | | | | | | |
| Intercept | 17.03 | 0.39 | 43.86 | 16.27 | 17.79 | < 0.001 |
| Density | -0.07 | 0.02 | -4.56 | -0.10 | -0.04 | < 0.001 |
| Age^2^ × forest cover × climatic subperiod | -2.35 | 0.66 | -3.56 | -3.64 | -1.05 | < 0.001 |
| **males** | | | | | | |
| Intercept | 17.42 | 0.38 | 46.13 | 16.68 | 18.16 | < 0.001 |
| Density | -0.12 | 0.02 | -5.05 | -0.15 | -0-08 | < 0.001 |
| Age^2^ × forest cover × climatic subperiod | -4.72 | 1.03 | -4.57 | -6.74 | -2.69 | < 0.001 |
|  |  |  |  |  |  |  |
